# Evolution characteristics of micromechanics provides insights into the microstructure of pharmaceutical tablets fabricated by bimodal mixtures

**DOI:** 10.1038/s41598-023-47239-w

**Published:** 2023-11-20

**Authors:** Mengtao Zhao, Anqi Luo, Yu Zhou, Zeng Liu, Yuting Wang, Linxiu Luo, Yanling Jiang, Jincao Tang, Zheng Lu, Tianbing Guan, Libo Chen, Huimin Sun, Chuanyun Dai

**Affiliations:** 1 Chongqing Key Laboratory of Industrial Fermentation Microorganisms, College of Chemistry and Chemical Engineering, Chongqing University of Science and Technology, Chongqing, 401331 China; 2 NMPA Key Laboratory for Quality Research and Evaluation of Pharmaceutical Excipients, National Institutes for Food and Drug Control, Beijing, 100050 China

**Keywords:** Chemistry, Engineering, Mathematics and computing

## Abstract

This research focuses on the evolution of mechanical behavior of bimodal mixtures undergoing compaction and diametrical compression. The clusters were built and discrete element method (DEM) was used to investigate the densification process and micromechanics of bimodal mixtures. Additionally, a more comprehensive investigate of the respective breakage of the bimodal mixtures has been carried out. On this basis, qualitative and quantitative analysis of the compressive force, force chain, contact bonds and density field evolution characteristics of the clusters are investigated during the compression process. The entire loading process of the clusters is divided into three stages: rearrangement, breakage and elastic–plastic deformation. Additionally, there are differences in the evolution of micromechanics behavior of different particles in the bimodal mixture, with pregelatinized starch breakage and deformation occurring before microcrystalline cellulose. With the tablet deformation, the fragmentation process of the tablet started at the point of contact and extended toward the center, and the curvature of the force chain increased. This approach may potentially hold a valuable new information relevant to important transformation forms batch manufacturing to advanced manufacturing for the oral solid dosage form.

## Introduction

Pharmaceutical tablets is the most widely used pharmaceutical product, making up more than 70% of all solid dosage forms, because of these benefits such their ease of use, manufacturing simplicity, and long shelf life^1^. Pharmaceutical tablets are generally produced by compacting the multi-component pharmaceutical mixtures, including active drugs and excipients. It is well recognized that properties of ingredients can affect the performance of the final tablet^2^. These properties include both physicochemical and mechanical properties and dictate how formulations will behave during tablet processing. Therefore, to design and develop a successful tablet having good performance such as tensile strength and release kinetics, it is critical to understand the evolution characteristics inside tablet during compression^3,4^. A vast exiting literature have been reported to grasp deeply the evolution of tablets. However, most of these research are limited to the description of experimental phenomena with long cycle time and high cost^5^. In addition, this research cannot truly describe the mechanism of inter-particle adhesion force formation during tablet compression, nor can truly insight the network distribution and strength of inter-particle force chains.

With the rapid development of various advanced technology, scholars have developed a series of theories and setups to understand the past (particles) and present (properties) of tablets. Paul adopted machine learning and multivariate instrumental analysis to investigate the correlation between tablet top cracking and pressure level, radial stress transfer, and mechanical properties of excipients to establish a tablet prediction model^6^. Meynard established a reliable analytical model based on Impulse Excitation Technique (IET) and finite element method (FEM) to investigate the correlation between different shapes and sizes of tablets and the occurrence of quality defects and the results indicate that special-shaped tablets are more easily to occur with quality defects^7^. However, due to the complex composition and diverse appearance structure of tablets, the methods above are also unable to reveal essentially the root reasons causing the quality differences. Therefore, the numerical simulation methods are necessary to use to explore the micromechanical mechanism of the tablet densification process.

The discrete element method (DEM)^8^ can directly inscribe the micromechanical response properties of granular systems from the particle scale, which plays a powerful role in revealing the microstructure and micromechanics of granular materials^9^. DEMs were initially used mainly for strain-softening and hardening^10^, local shear failure^11^, shear-induced anisotropy^12^ of large granular materials such as soils and rocks. The DEM will be used in the densification process and the compaction molding mechanism of particles as it received further development^13,14^. Recently, the DEM considering the attractive inter-particle forces, e.g., adhesion and van der Waals interaction^15^. This provides the possibility for DEM to simulate the compaction process of micron-sized particles or powders. However, when the external loads are applied to the multi-component pharmaceutical mixtures, the original particles fragment into smaller-sized particles. The bonded particle model (BPM), which creates non-overlapping clusters of solid spheres by solid bonding, allows for the simulation of cohesive damage and microscopic deformation^16,17^. In addition, under the influence of the external compression, the particle mixture creates an irregular force chain network for transmitting the majority of the external load^18^. The force chain network is unique and historic due to friction^19^. After tablet formation, the force chains between particles at this time include, but are not limited to, hydrogen bonding, van der Waals bonding, and solid bridge formation^20^. DEM can capture these micromechanical behavioral features from the particle scale. Gou^21^ investigated the effect of particle breakage on particle compaction by DEM, and the breakage mechanisms of compacts was analyzed in terms of force chains and energy distribution. However, before the potential of any of the above applications can be fully achieved, there remains much work to done understanding the micromechanical mechanism inside tablet during processing. And few attempts have been made to simulate the breakage and densification behavior of bimodal mixture particles during compaction and diametrical compression using DEM, especially for micron-sized pharmaceutical excipients.

To obtain a better understanding of the microstructure of pharmaceutical tablets from evolution characteristics of micromechanics, the pharmaceutical tablets are fabricated by bimodal mixtures including pregelatinized starch (PGS) and microcrystalline cellulose (MCC), an elastic excipient and a plastic excipient, respectively. The DEM parameters of PGS and MCC are first determined, this part is based on previous laboratory work and is not investigated in this paper. Then, a bimodal mixtures tablet compression model was constructed, and the material particles were represented by clusters to reveal the microstructure and micromechanical evolution of the particles during the tablet compression and diametrical compression processes. Finally, a special-shaped cluster model was constructed. The inter-particle compressive force, force chain, density field and coordination number during compression to reveal the mechanism by which different special-shaped particles affect the quality control of tablets. This research will provide a scientific reference for the selection of reasonable excipients in formulation development and provide reliable data to support the establishment of more scientific quality standards.

## Materials and methods

### Simulation of the compression test

#### Contact model

**Figure 1:**
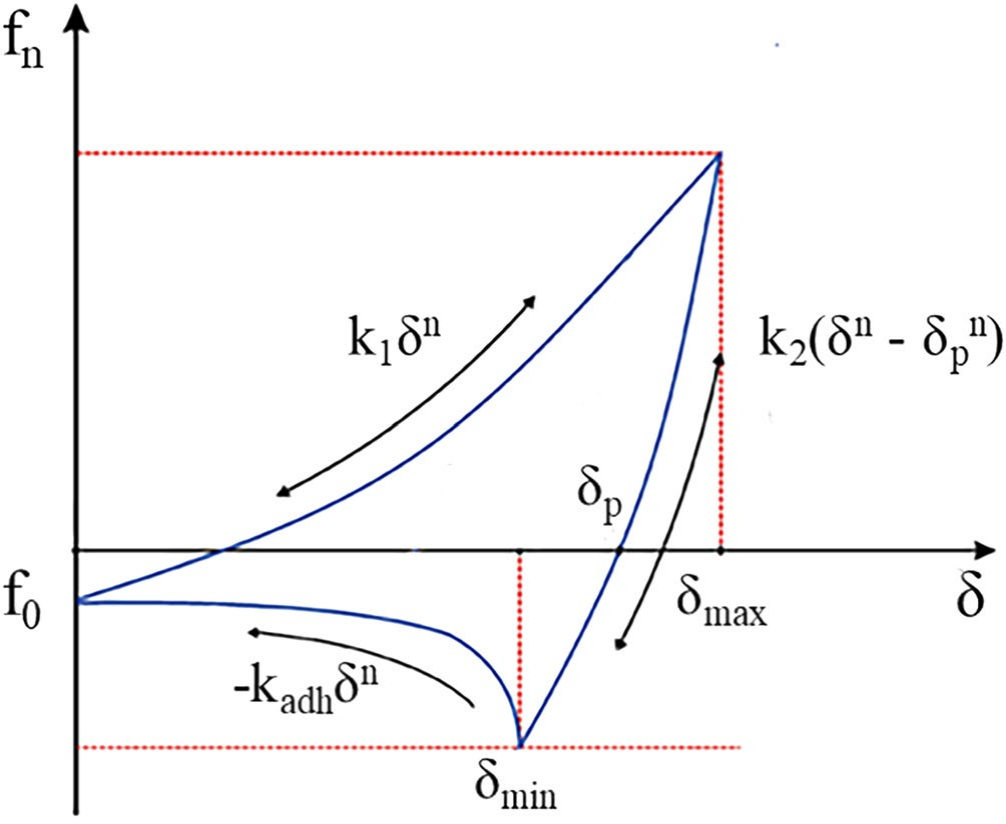
Normal contact force–displacement function of EEPA base model.

DEM was used in the numerical simulations, due to the peculiarities of the compression process, many compressed granular materials exhibit elastic–plastic deformation accompanied by an increase in cohesive forces at the mesoscopic scale. Therefore, the Edinburgh Elasto-plastic Adhesion (EEPA) model established by the University of Edinburgh is used in this research^22^. The schematic diagrams of particle contact and normal force-overlap (f_n_ − δ) curve for this model are shown in Fig. 1. It is a multifunctional nonlinear model that contains hysteresis, cohesion and van der Waals-like forces in the contact mechanics equations.

Where k_1_ is the loading stiffness parameter (kN/m), k_2_ is the unloading stiffness parameter (kN/m), k_adh_ is the adhesive stiffness parameter (kN/m), δ is the total normal overlap (m), δ_max_ is the maximum normal overlap (m), δ_p_ is the plastic overlap (m), f_0_ is the Constant adhesive strength at first contact (N) and n is the stiffness exponent.

#### Construction of clusters

**Figure 2:**
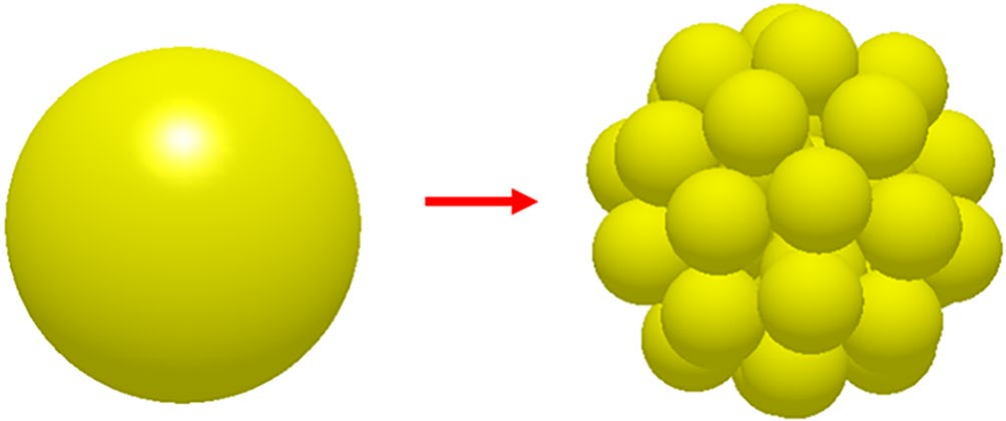
Schematic diagram of particle cluster.

In the compression simulation process, particle deformation is considered, and its mechanical properties are more complex. When constructing the clusters, here the primary solid spheres are equivalent to the PGS and MCC in size, 50 μm (the particle sizes were measured using a Winner 319B particle size analyzer), and the radius of the clusters body is set to 0.4 mm (Fig. 2), which is chosen based on the pre-experimental results and ultimately used for accelerated simulation. On this basis, special-shaped particles and special-shaped clusters were constructed. Here, the special-shaped particles are sub-particles formed by the overlapping of a single or multiple spheres. The special-shaped clusters are composed of single spherical particles directly combined. Then, the compaction and diametrical compression models of special-shaped particles and special-shaped clusters were constructed to investigate the mechanism of their influence on the micromechanics behavior of the tablets.

#### DEM parameters

It is worth noting that the intrinsic parameters of the particles in the compression experiments can significantly affect the formation of the tablet. First, the DEM parameters of PGS and MCC were obtained using the optimal Latin hypercube sampling technique, and the compressive force-hardness curve and the compressive force-volume reduction curve produced from physical experiment and simulation were quantified, the results are shown in Tables S1 and S2, respectively. The results of the PGS physical experiment and simulation's compressive force-hardness curve and compressive force-volume reduction curve have been plotted and they are shown in Figs. S1 and S2, respectively. The results of the MCC physical experiment and simulation's compressive force-hardness curve and compressive force-volume reduction curve have been plotted and they are shown in Figs. S3 and S4, respectively. The accuracy of the Kriging model is then verified using 10 sets of sample points, and the verification results of PGS and MCC are represented in Figs. S5 and S6, respectively. Finally, the appropriate DEM parameters were determined using multi-objective genetic algorithm (NSGA-II). Therefore, the DEM parameters involved in the compression simulation process are listed (Table 1).

**Table 1:** DEM parameters for compaction and diametrical compression.

Parameter	Unit	PGS	MCC	Bimodal mixtures
Poisson's ratio	–	0.257	0.381	–
Shear modulus	MPa	1 × 10^9^	1.04 × 10^9^	–
Static friction coefficient	–	0.165	0.719	0.1
Rolling friction coefficient	–	0.208	0.294	9.95 × 10^9^
Normal stiffness per unit area	N m^−3^	2.42 × 10^9^	3.17 × 10^9^	1.99 × 10^9^
Shear stiffness per unit area	N m^−3^	7.95 × 10^9^	6.75 × 10^9^	− 0.0604
Constant pull-off force	N	− 0.0092	− 0.0387	–
Contract plasticity ratio	–	0.8	0.9	0.85

#### Compaction test

**Figure 3:**
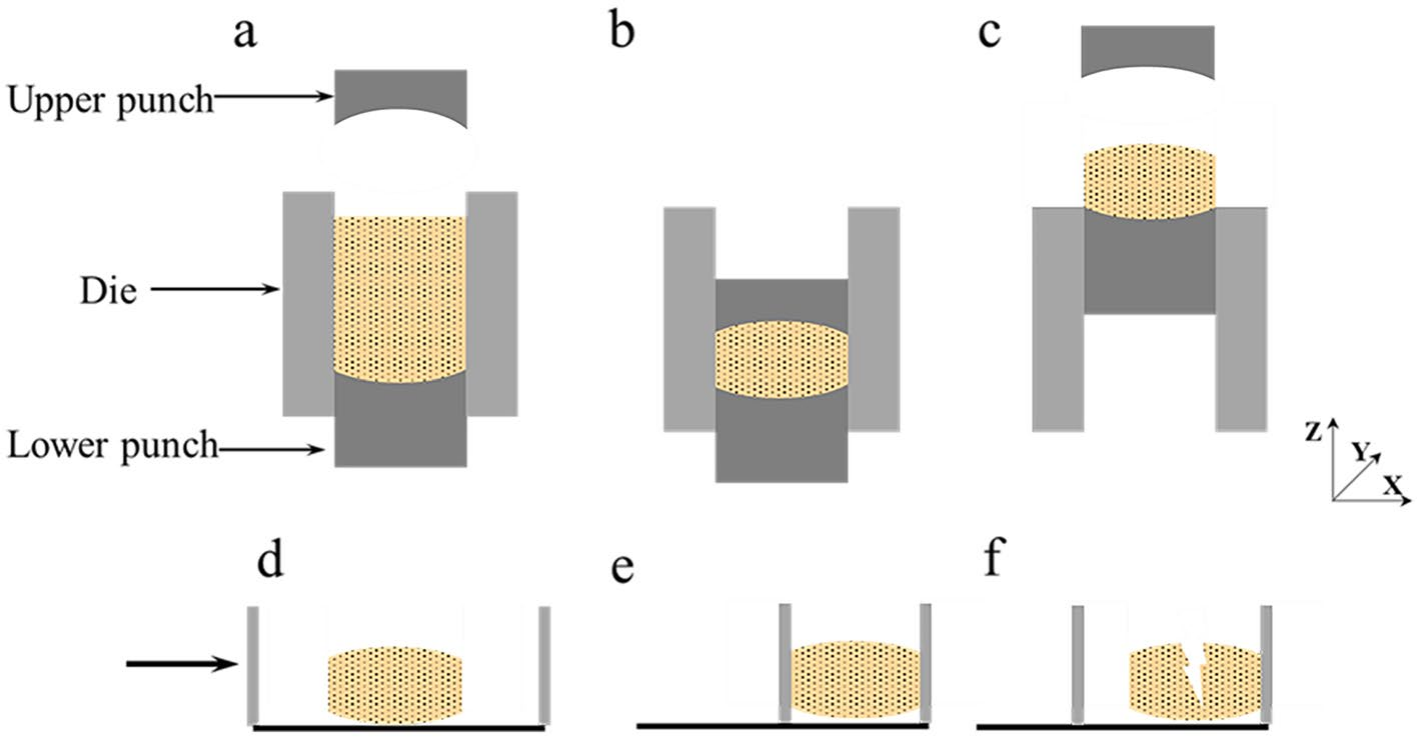
Numerical simulation of tableting and diametrical compression process: (**a**) Powder filling; (**b**) Compression; (**c**) Decompression; (**d**) The left loading plate starts to move; (**e**) The left loading plate first contacts the tablet; (**f**) Fragmentation of tablet.

Numerical simulations were performed to investigate the pressure of particles in a cylindrical device. Compaction test simulations were performed in an analogous manner as the corresponding experiments. Particle was generated randomly in the die (Fig. 3a). After settling owing to gravitation, the particles were compacted with upper punch to the assumed value of the thickness (Fig. 3b). After the desired thickness was reached, the upper punch was unloaded with the same velocity. Next, the tablet was separated from the device (Fig. 3c). Here, the tablet has a diameter of 2 mm and the curvature radius of the convex surface is 2.6 mm. Then, numerical simulations were performed to investigate the hardness of tablet in a diametrical compression device. Similarly, diametrical compression test simulations were performed in an analogous manner as the corresponding experiments. The tablet was placed in a diametrical compression device (Fig. 3d), and the left loading plate moved forward along the X-axis at a velocity of 0.0085 m s^−1^ (Fig. 3e) until tablet was broken (Fig. 3f).

### Micromechanical analysis

During tablet compaction, particles are subject to rearrangement, breakage, and elastic–plastic deformation due to external loading forces. The formation of an irregular contact network between the particles and the transmission of loading forces along the load point to the center, eventually forming a complete force chain network^18^. The research of the force chain spreading process and the change law is essential to investigate the compressibility of particles. Therefore, the evolution of the micromechanics (particle compressive force, force chains, contact bonds, and density fields) of bimodal mixtures during compaction and diametrical compression process was extracted in this paper. The mechanism of particle material impact on the microstructure and micromechanics of the tablets is revealed from the microscopic perspective.

## Results and discussion

### Analysis of micromechanics behavior of compaction process

**Figure 4:**
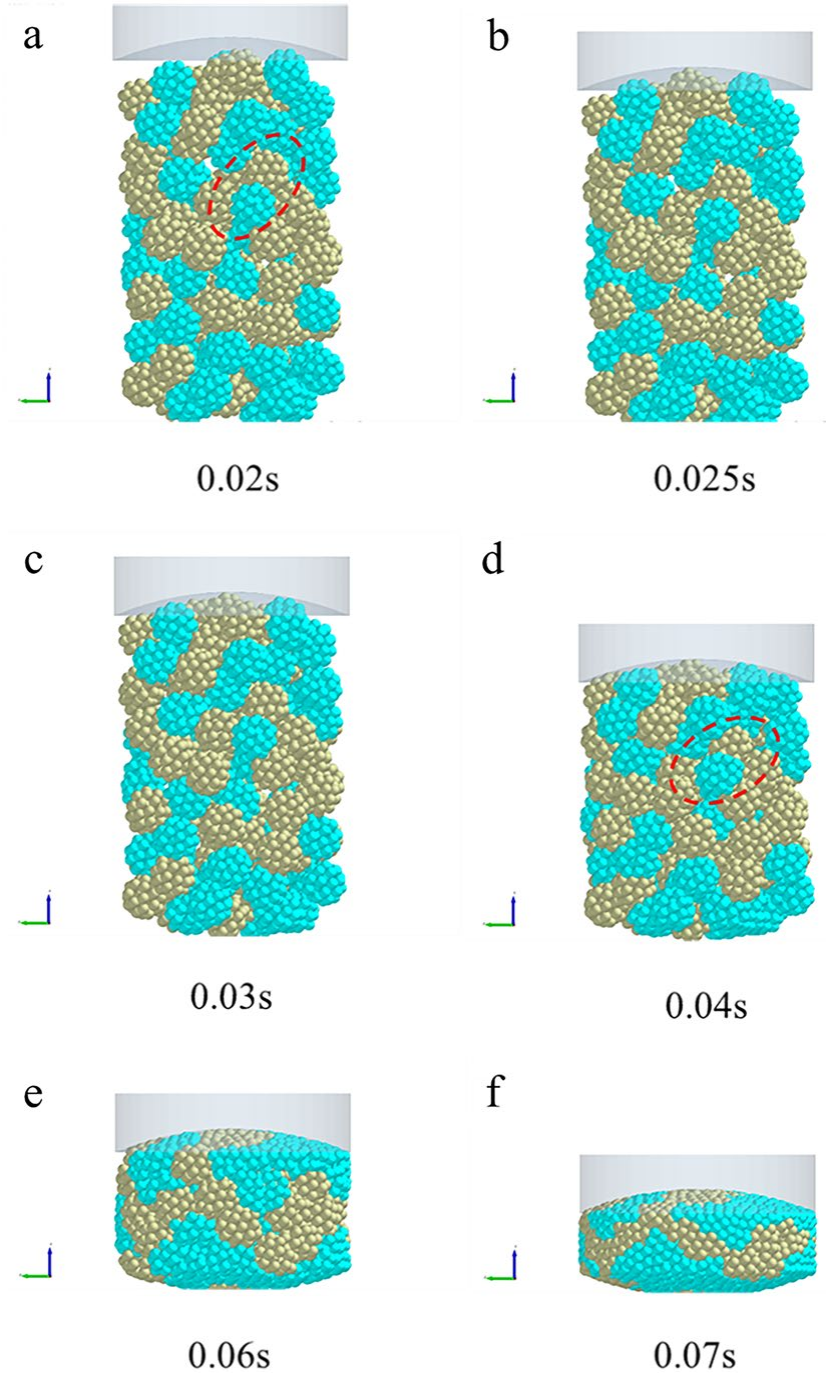
Analysis of cluster particle deformation during the compaction of bimodal mixtures.

**Figure 5:**
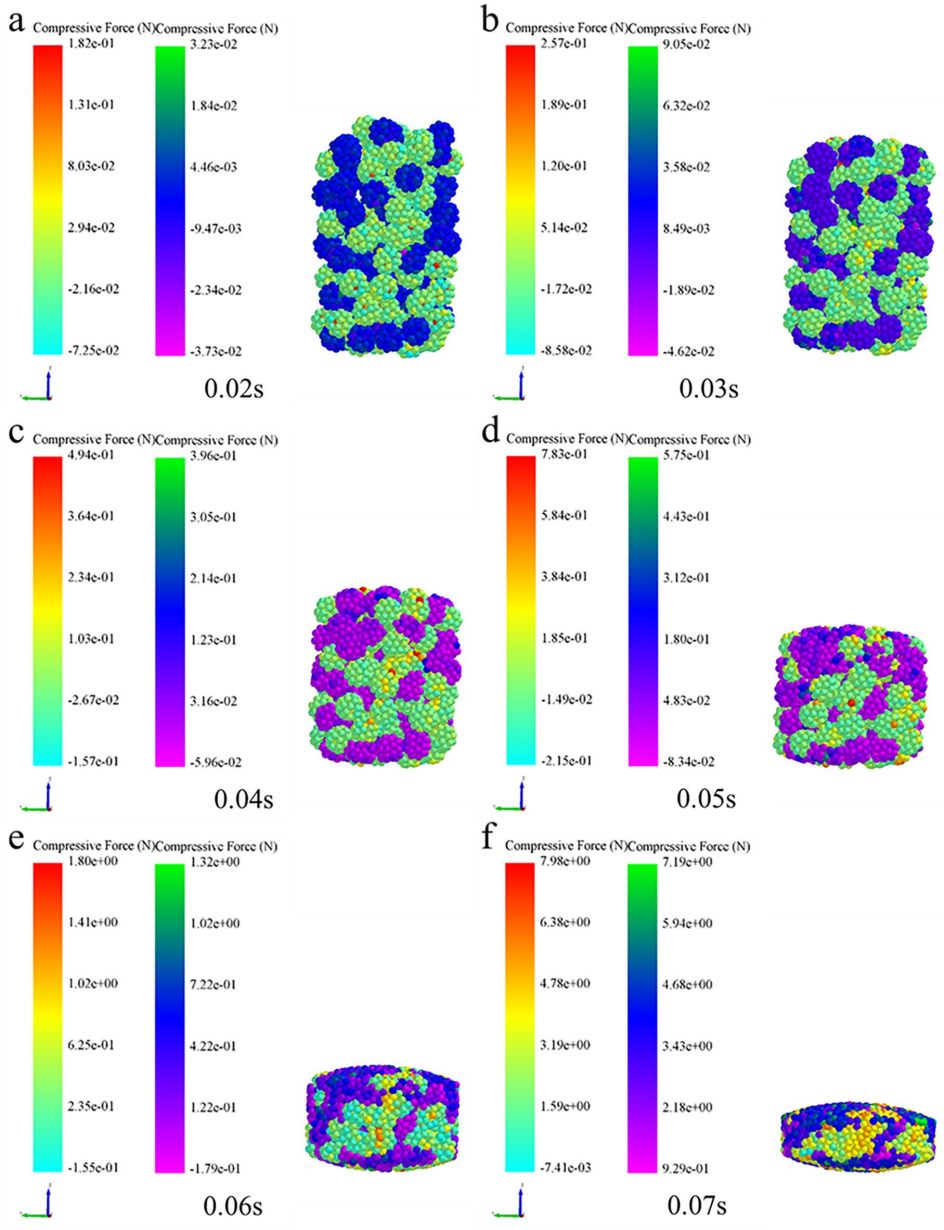
Analysis of cluster particle compressive force during the compaction of bimodal mixtures.

**Figure 6:**
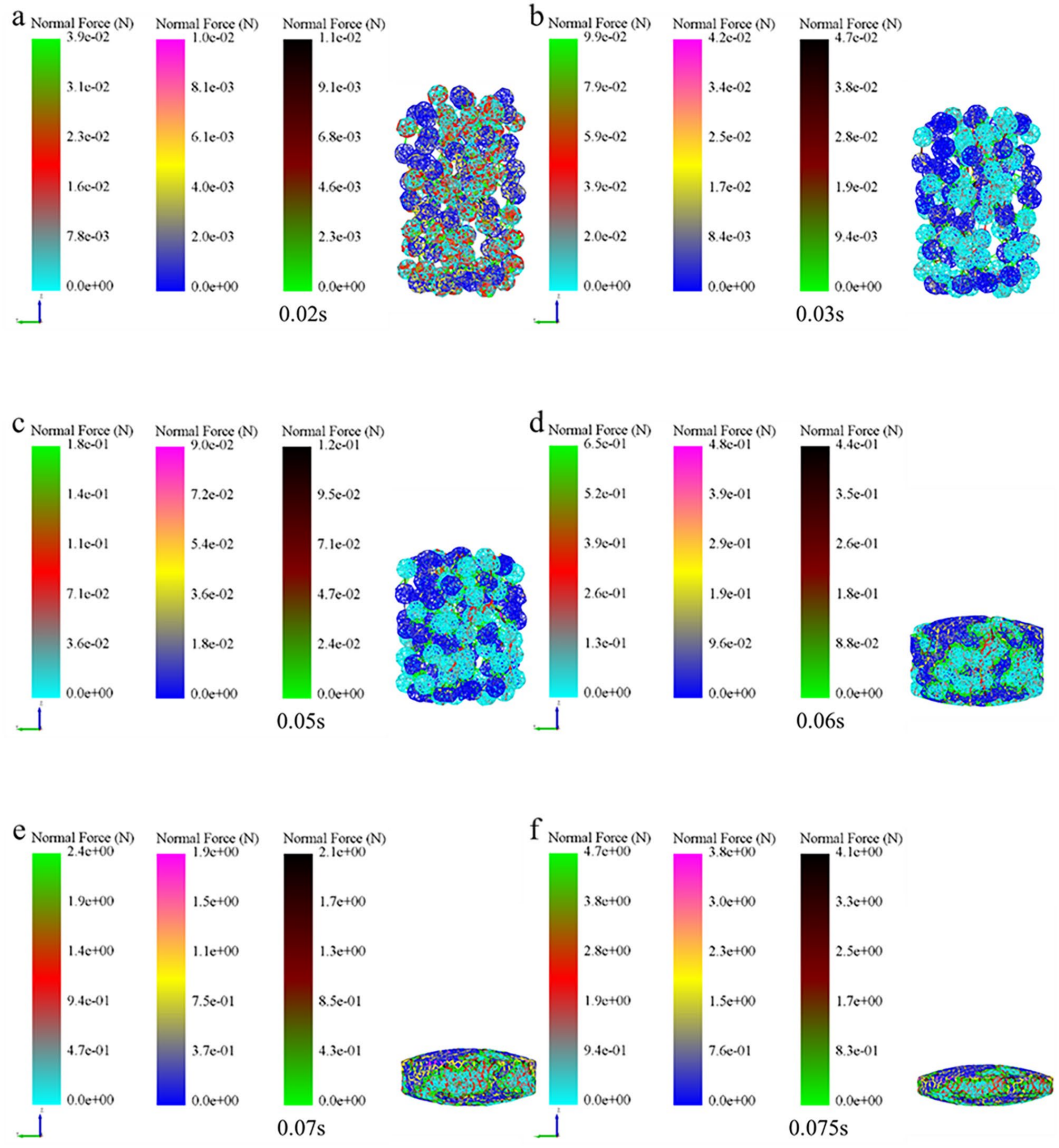
Analysis of force chain changes during the compaction of bimodal mixtures.

**Figure 7:**
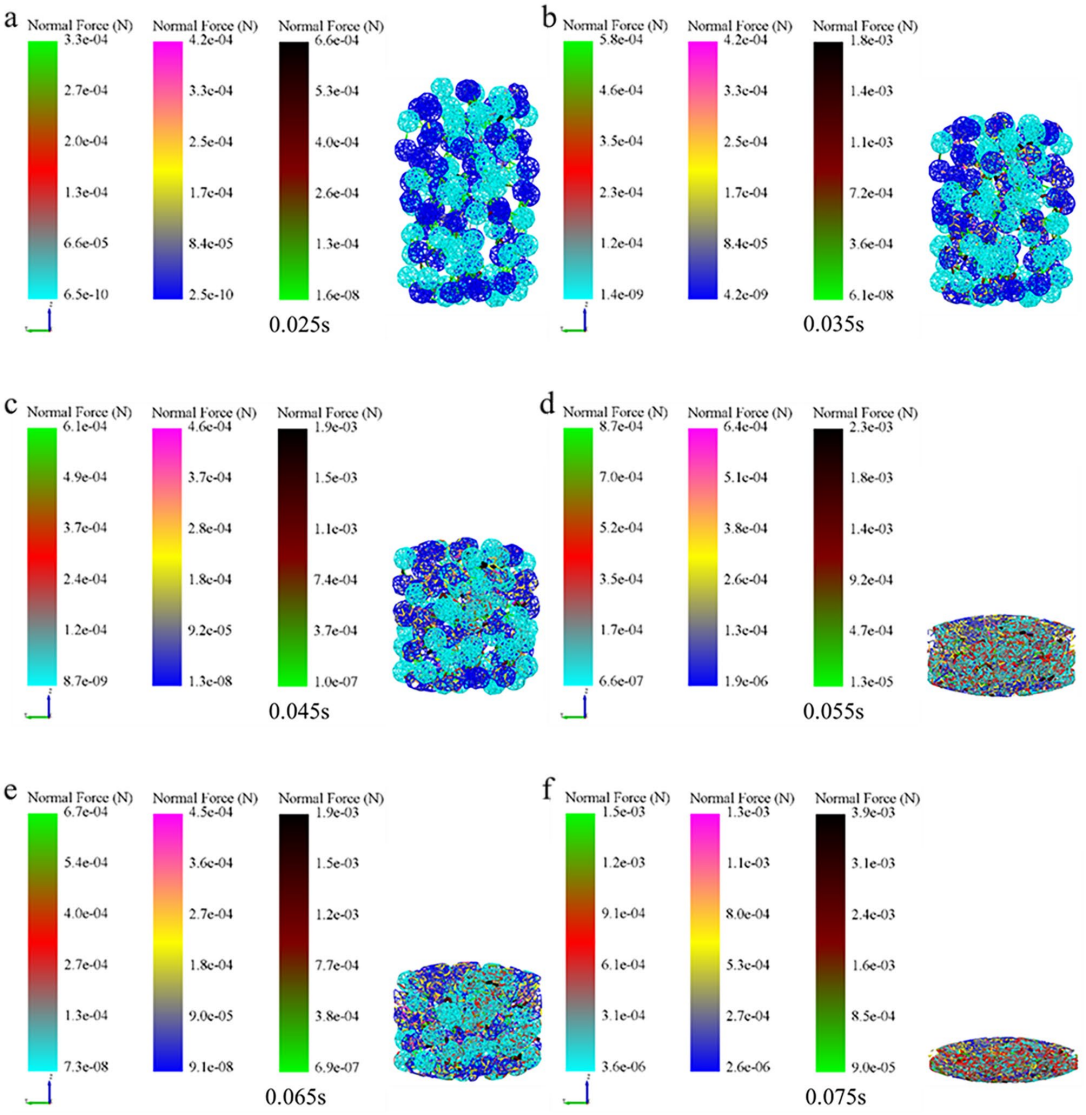
Analysis of contact bonds changes during the compaction of bimodal mixtures.

**Figure 8:**
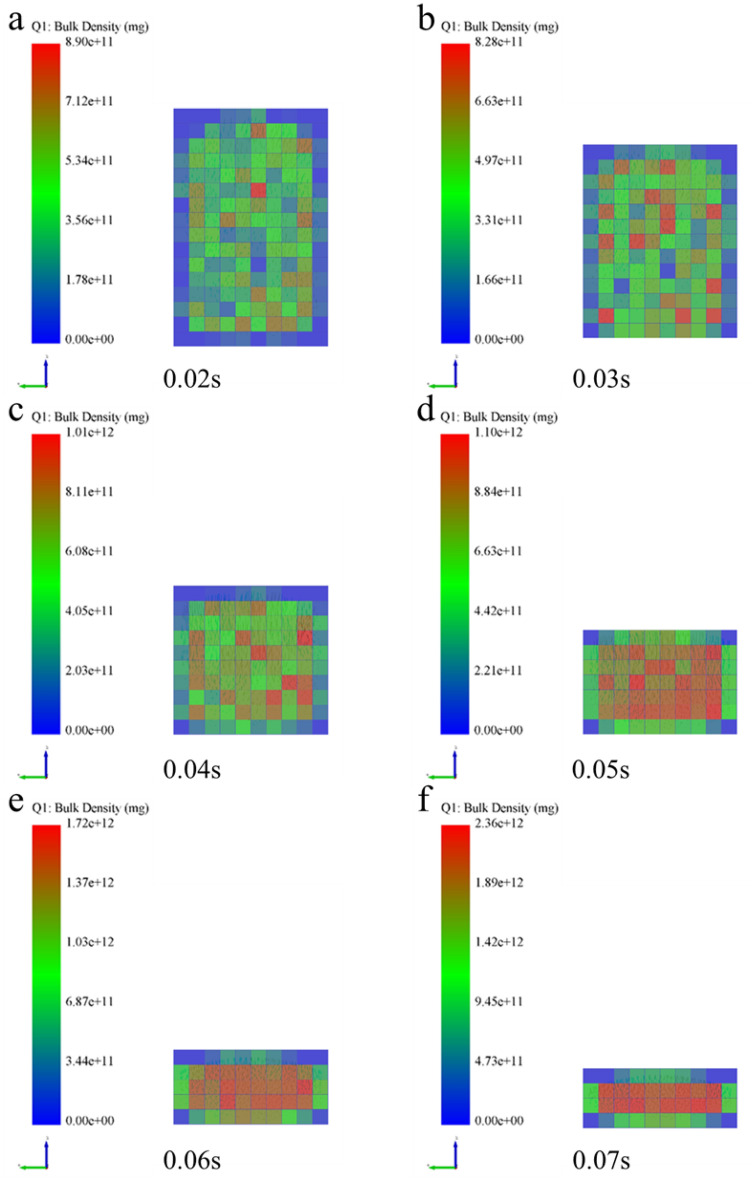
Analysis of density changes during the compaction of bimodal mixtures.

In order to further examine the formation mechanism of the micromechanics during the tablet-forming process, the changes in micromechanics of the particles during the compression process were extracted and presented in this work. According to the clusters shape (Fig. 4), compressive force (Fig. 5), force chain (Fig. 6), contact bonds (Fig. 7), and density (Fig. 8). The first legend in this research stands for MCC, the second legend for PGS, and the third legend for bimodal mixtures, except for density. It can be clearly seen that in the rearrangement stage of pressure loading, clusters are closer to each other, and the gaps between clusters are slowly filled. At this stage, there is no significant breakage of clusters (Fig. 4a–c), less compressive force on clusters (Fig. 5a,b), force chains between clusters (Fig. 6a,b), no significant broken of contact bonds (Fig. 7a,b), and no significant change in density field (Fig. 8a,b). At this stage, the porosity of the whole particle system is affected by the friction between particles^23^. In the breakage stage, the compressive force increases and the clusters breakage (Fig. 4d,e), and mostly at the top of the particle system (Fig. 5c–e), which is caused by the continuous increment of external loading force, the clusters were breakage because they cannot bear the external load^24^. The force chains extended from the top to the bottom of the tablets, parallel to the direction of the loading force, and the strong force chains were transferred from within the clusters to between the clusters (Fig. 6c,d). This was demonstrated in a research by Tian^25^, where the force chains had no specific orientation in the initial state, and when they added load to the system, the direction of the force chains rapidly shifted from non-oriented to the loading direction with the onset of axial strain, and were always mainly parallel to the loading direction. The contact bonds broken is evident (Fig. 7c–e), and aggregation of particles occurs in some regions (Fig. 8c–e). At this stage, clusters breakage and fragments appear to fill the gaps between clusters, at which point the rate of porosity reduction accelerates. In the whole compression process, the compressive force and density reach the maximum at elastic–plastic deformation stage. At this stage, the loading compressive force increases sharply, the particle system is concentrated in a narrow region (Fig. 8f). The clusters breakage was replaced, and the particles undergo elastic–plastic deformation^26^. The particle fragments overlapped with other particles, so as to form an interaction force between particles (consolidation or solidification of liquid film, Van Der Waals force, ionic bond and covalent bond), and finally form a dense solid tablet.

**Figure 9:**
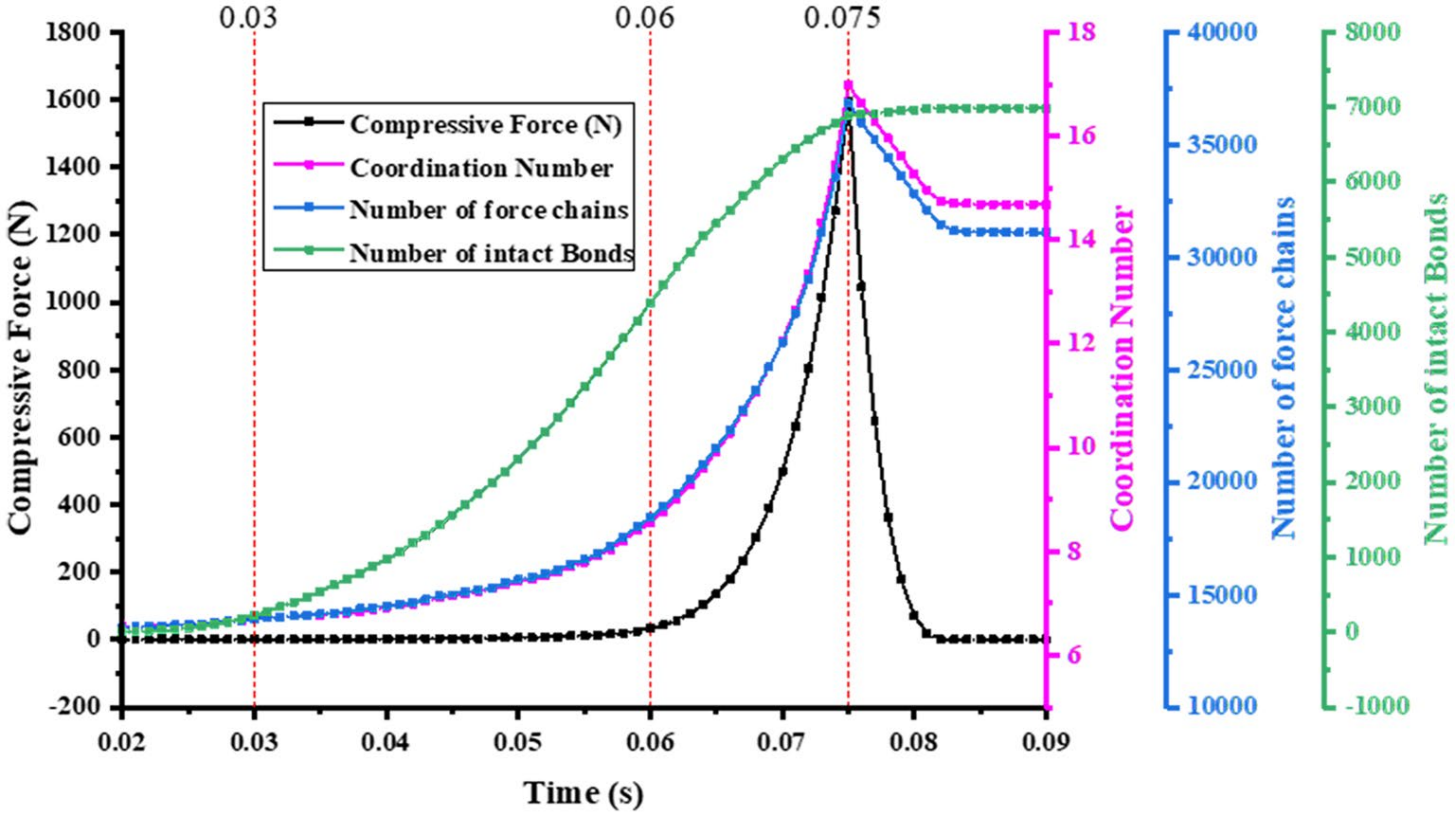
The relation curve between micromechanics behavior and time of the bimodal mixtures.

Figure 9 shows the compressive force–time curve, coordination number-time curve、number of force chains-time curve and number of intact bonds-time curve during the compaction of the bimodal mixtures. The curves in Fig. 9 can be divided into three stages: the rearrangement stage (0.02 s–0.03 s), the breakage stage (0.03 s–0.06 s) and the elastic–plastic deformation stage (0.06 s–0.075 s). It can be seen from Fig. 9 that the rearrangement stage exhibits a limited increase in the Compressive Force、Coordination Number、Number of force chains and Number of intact Bonds. In the breakage stage, the breakage of clusters leads to a decrease in the number of contact bonds. The increase in inter-particle contact leads to an increase in both the number of force chains and the coordination number. In the elastic–plastic deformation stage, the particle deformation occurs as a result of compression, and the particle system porosity decreases sharply. At this time, the compressive force, number of force chains and coordination number are exponentially increased.

**Figure 10:**
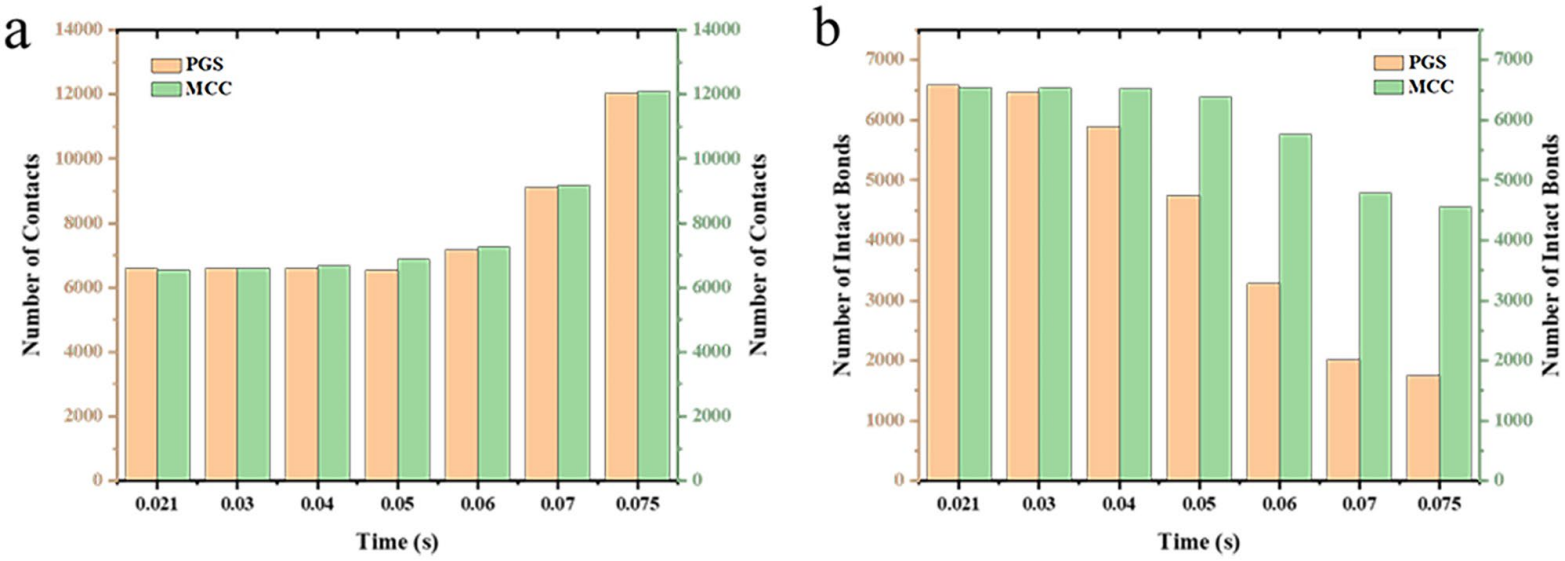
Comparison of the number of force chains and contact bonds.

To gain a more comprehensive understanding of the respective breakage of the bimodal mixtures, the evolution of the compressive force, force chains, and contact bonds at the characteristic points were enlarged and scrutinized in Table 2. At the initial stage, the clusters are less stressed, and both PGS (blue) and MCC (cyan) clusters remain in their original state, and the strength of force chains and contact bonds inside the clusters is higher for MCC than PGS. At the rearrangement stage, the clusters were mainly rearrangement, and the increase in the number of force chains was not significant (Fig. 10a). In the breakage stage, when the loading force exceeded the preset critical stress value, the contact bonds between the clusters were broken. At this stage, the PGS clusters breakage first and the MCC clusters remained original. The evolution of contact bonds could evidence the above results, the contact bonds inside the PGS clusters started to broken first and the trend of contact bonds inside the MCC clusters was not obvious. With the continuous loading of external forces, the MCC clusters gradually breakage, and the contact bonds within the clusters start to broken (Fig. 10b). This can be explained by the elastic–plastic nature of the different excipients. MCC, as a plastic excipient, is distinguished from the elastic excipient PGS by its compressibility. During direct compression, the PGS particles undergo elastic deformation after being stressed, which is accompanied by elastic recovery, which leads to the susceptibility of the PGS inter-particle force chain to broken^27^.

**Table 2:**
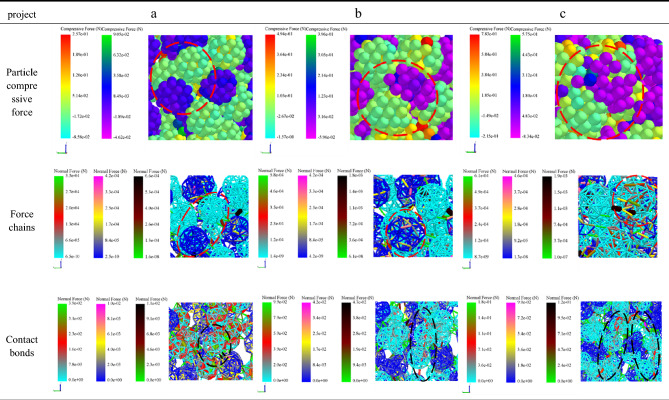
The evolution of particle compressive force, force chains and contact bonds at characteristic points during the compaction of bimodal mixtures.

### Analysis of micromechanics behavior of diametrical compression process

**Figure 11:**
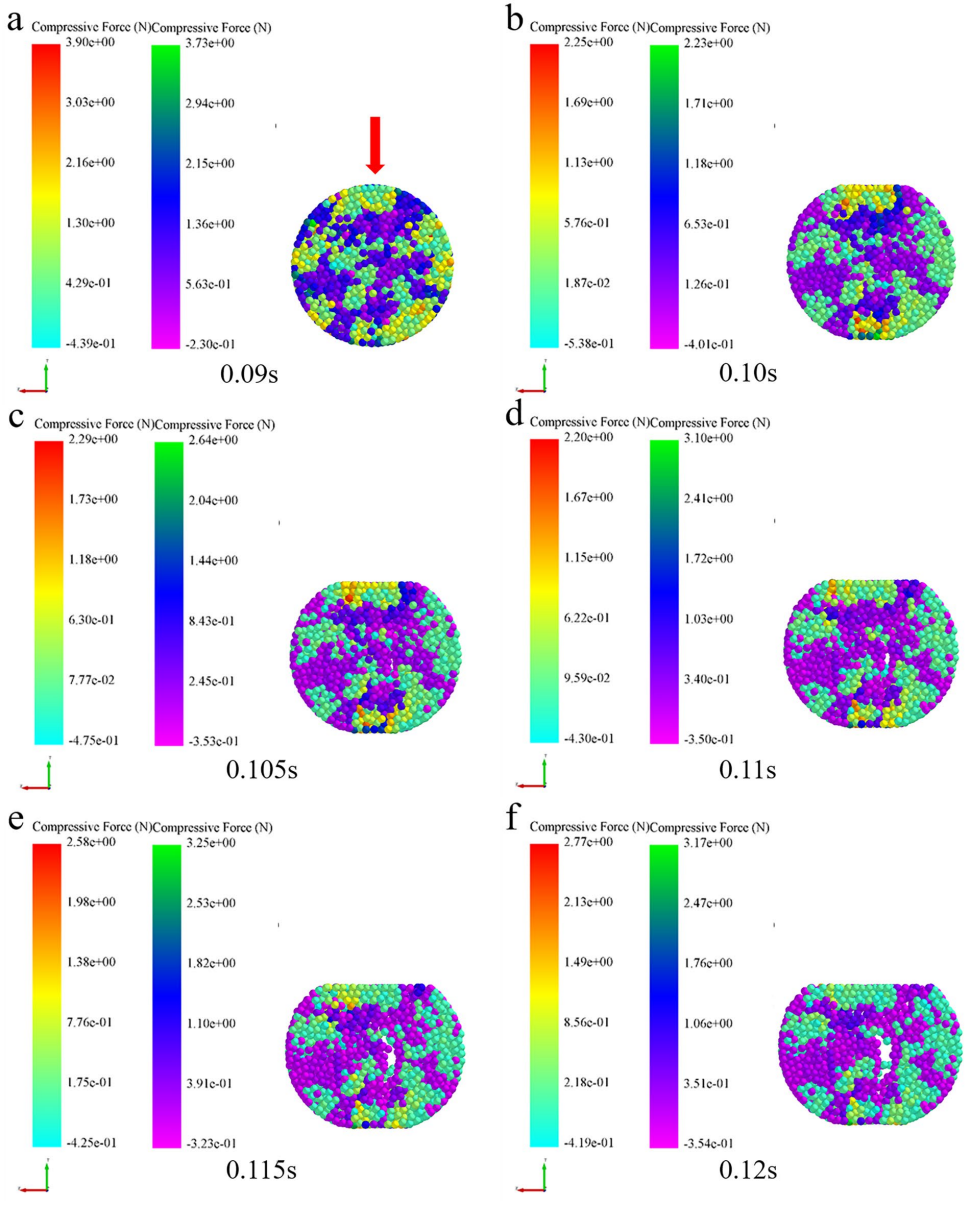
Analysis of particle compressive force during the diametrical compression of bimodal mixtures.

**Figure 12:**
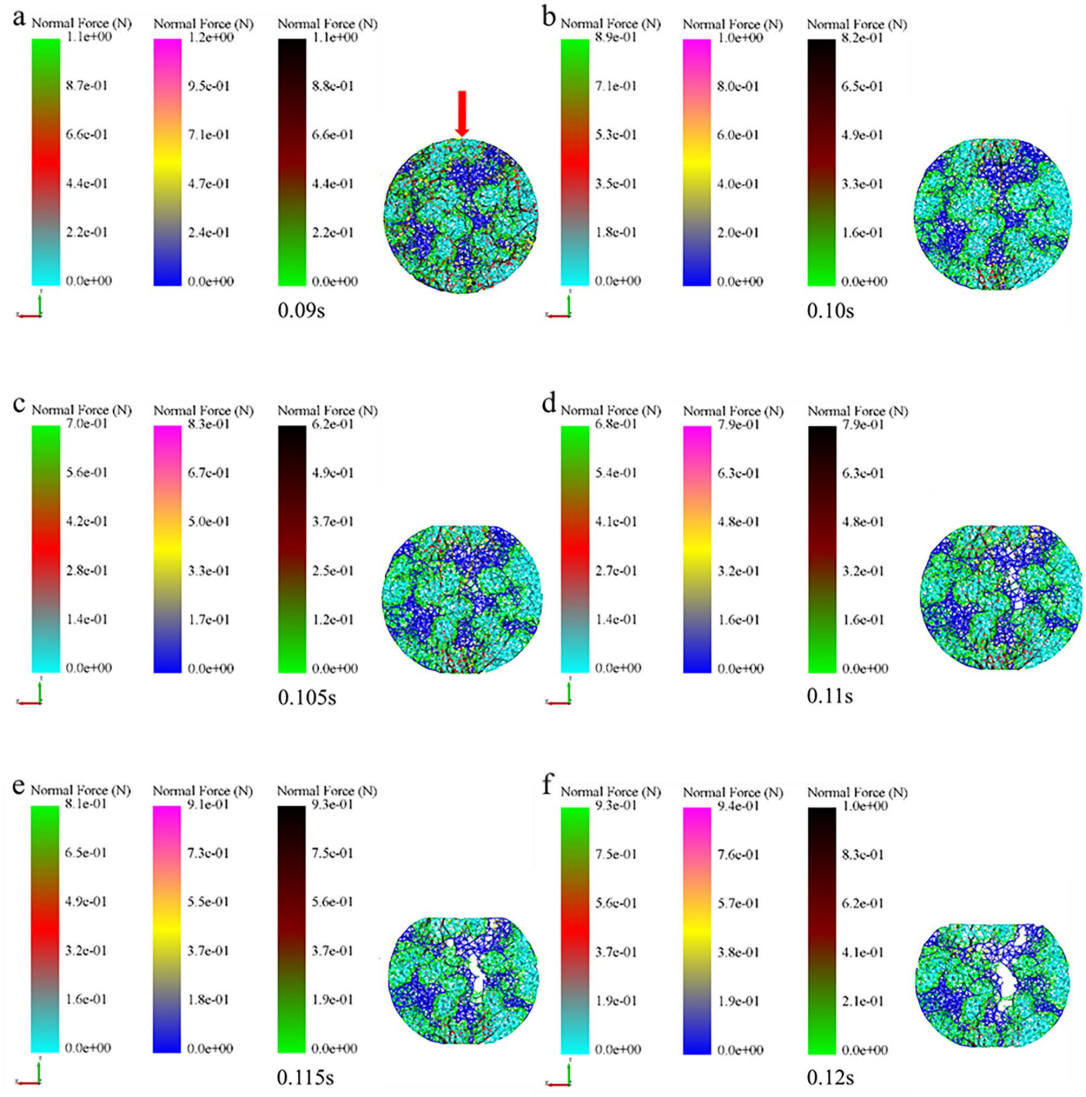
Analysis of force chain during the diametrical compression of bimodal mixtures.

**Figure 13:**
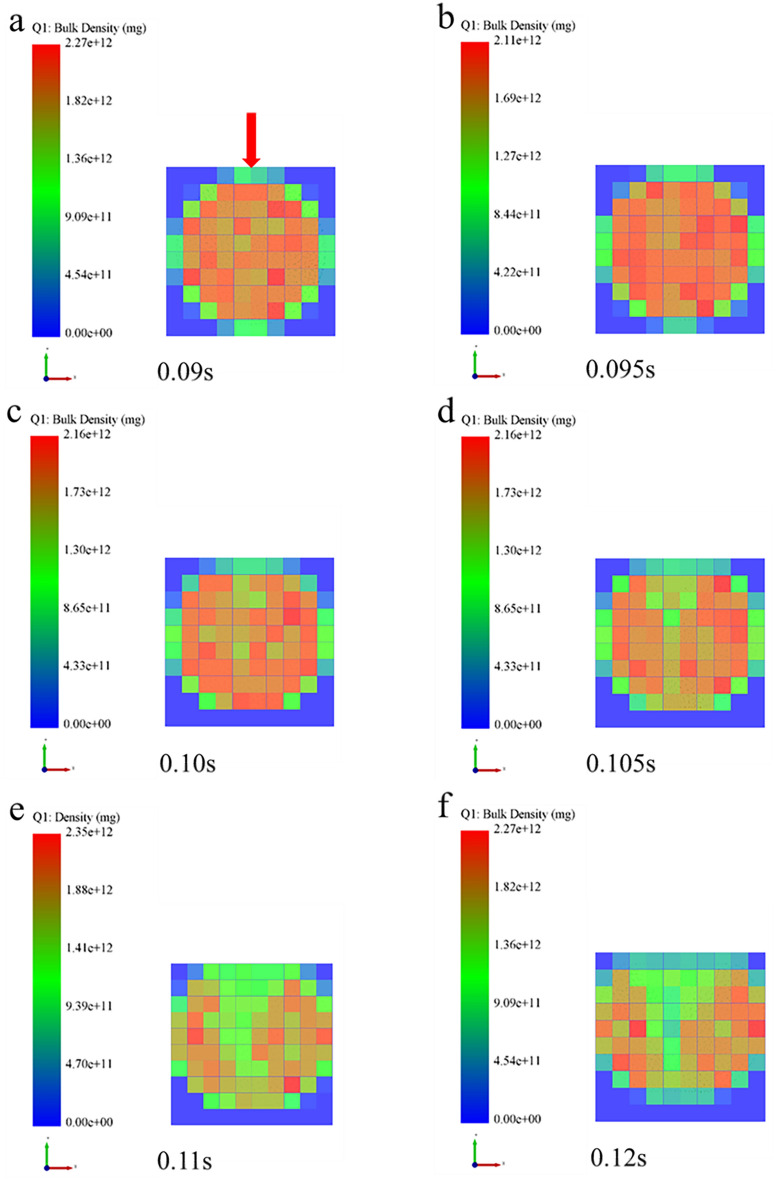
Analysis of density during the diametrical compression of bimodal mixtures.

Extracting the compressive force (Fig. 11), force chain (Fig. 12) and density (Fig. 13) among the inter-particle in the diametrical compression process of bimodal mixtures tablets, the legend is consistent with the compaction process. It was evident that the strong force chains began to emerge at the loading point and progressively concentrated into two symmetrical arch-shaped chains (Fig. 11). As the tablet deformed, the curvature of the strong force chains increased (Fig. 12), cracks emerged near the center of the tablet and expanded along the direction perpendicular to the loading plate, broken the connection between the inter-particle force chains (Figs. 11e, 12e). Fahad investigated the changing pattern of the mechanical behavior of gypsum discs during diametrical compression and found that the damage of the discs was due to shear and compressive stresses at the loading point^28^. In the diametrical compression test, the direction of the force chain is parallel to the direction of the loading force and extends to the center of the tablet. This phenomenon is consistent with the conclusion that the direction of the force chain during the compaction process is parallel to the loading direction of the force. This is consistent with Horabik's research results that during diametrical compression, the force chain between potato starch granules gradually divides into two symmetrical arched chains, and with the continuous deformation of the tablet, the curvature of the force chain increases and cracks appear most initially in the center of the tablet and extend along the vertical direction toward the loading plate^29^. This phenomenon can be explained by the change of density field during diametrical compression test. At this point, the particles move outward and the central density of the tablet decreases (Fig. 13c,d). Consequently, because of the force and its opposite reaction, the immovable pressing plate will apply an equal and opposite pressure to the particle system during pressurization. The pressing plate's motion prevents the particles from transitioning to an area of low density, so they must travel only in the direction perpendicular to the platen movement. The particles in the radial area of the tablet gradually decrease, resulting in fragmentation.

### Analysis of micromechanics behavior of special-shaped clusters

**Figure 14:**
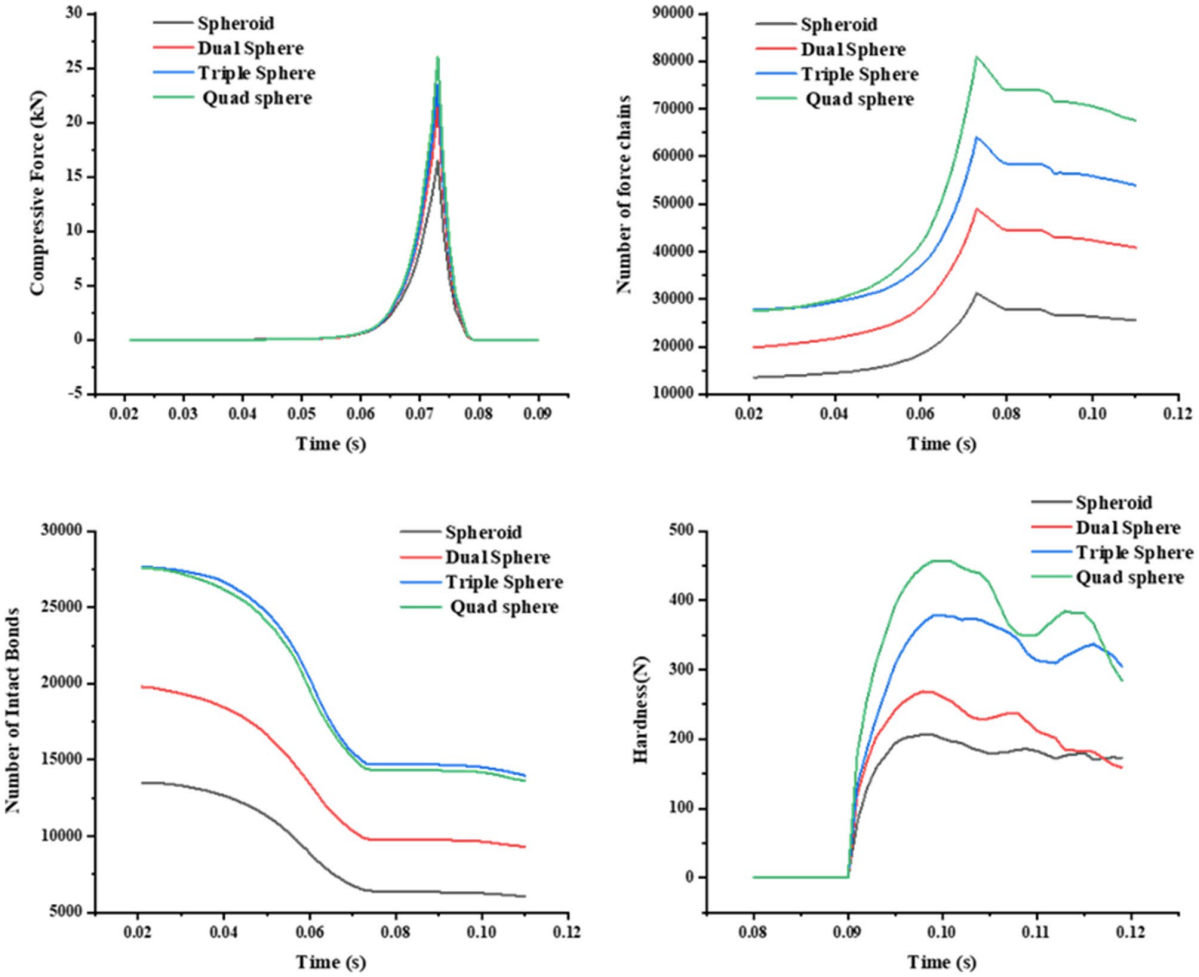
The relation curve between micromechanics behavior and time of the clusters formed by special-shaped particles.

**Figure 15:**
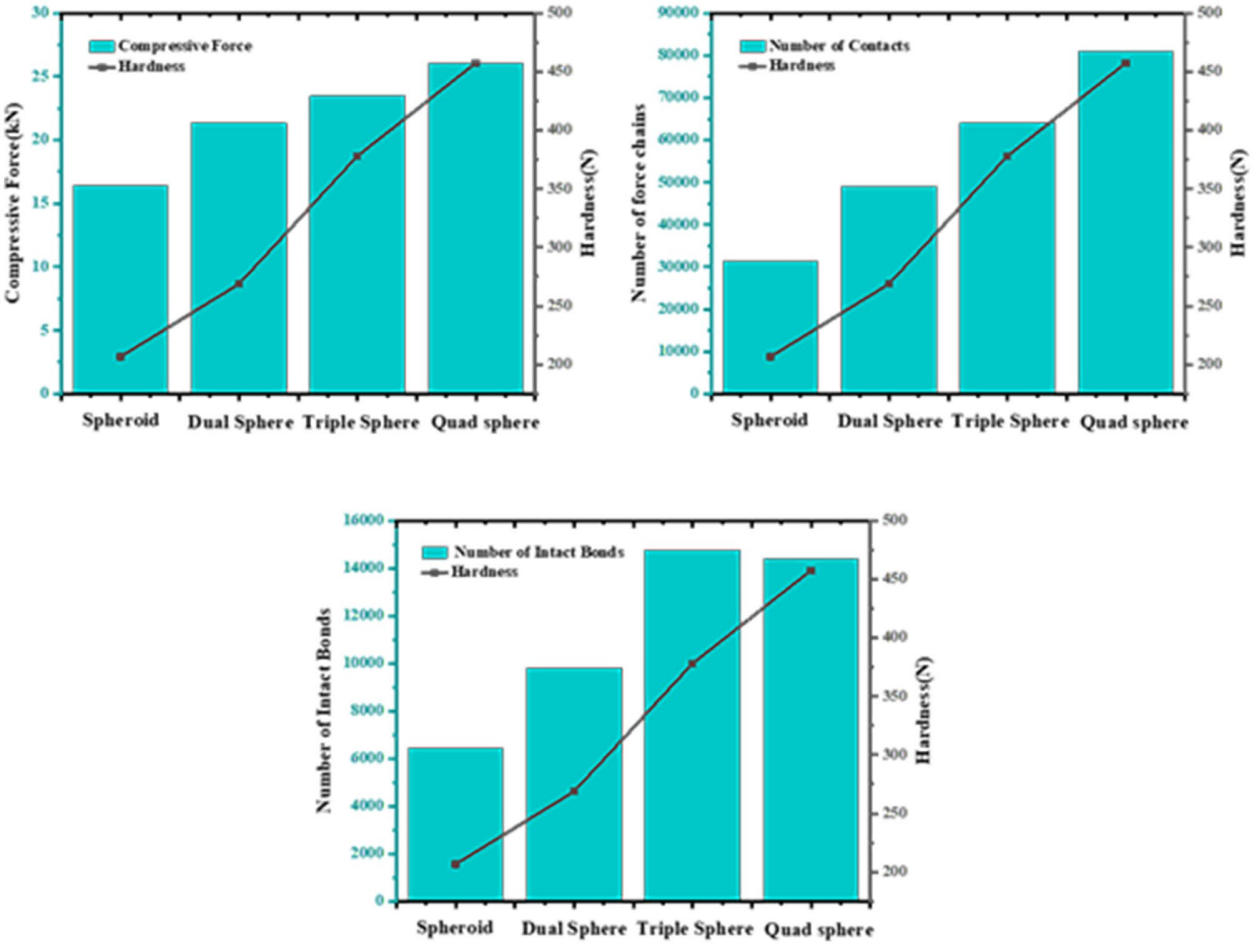
The compressive force, force chain and contact bonds of clusters formed by special-shaped particles in relation to tablet hardness at characteristic time points.

This research further explored the mechanism of the impact of special-shaped particles (quad spheres, triple spheres, dual spheres, and spheroid) on the micromechanics of the tablets (Table 3). The curves of compressive force, force chain, contact bonds and hardness with time for tablets formed by different shaped particles were extracted (Fig. 14). In addition, the relationship between compressive force, force chain and contact bonds at characteristic time points and tablet hardness was investigated (Fig. 15). The results indicate that the hardness, compressive force, force chain strength and contact bonds strength are proportional to the number of single spheres in the shaped particles, i.e., quad spheres > triple spheres > dual spheres > spheroid. The reason is that multiple contact is more likely to happen with the increase of the number of component particles, and the contact area of quad spheres are all larger than others. The fragments of clusters are more likely to fill the gaps between particles, making it easier to form mechanical interlocks between particles^30,31^. This is one of the reasons for the higher stiffness of the tablets formed by quad spheres particle clusters.

**Table 3:**
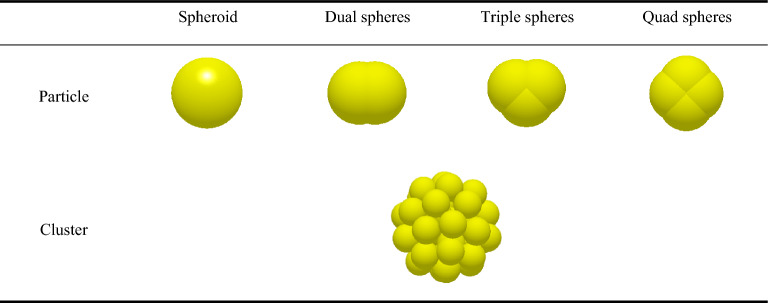
Clusters built by special-shaped particles.

**Figure 16:**
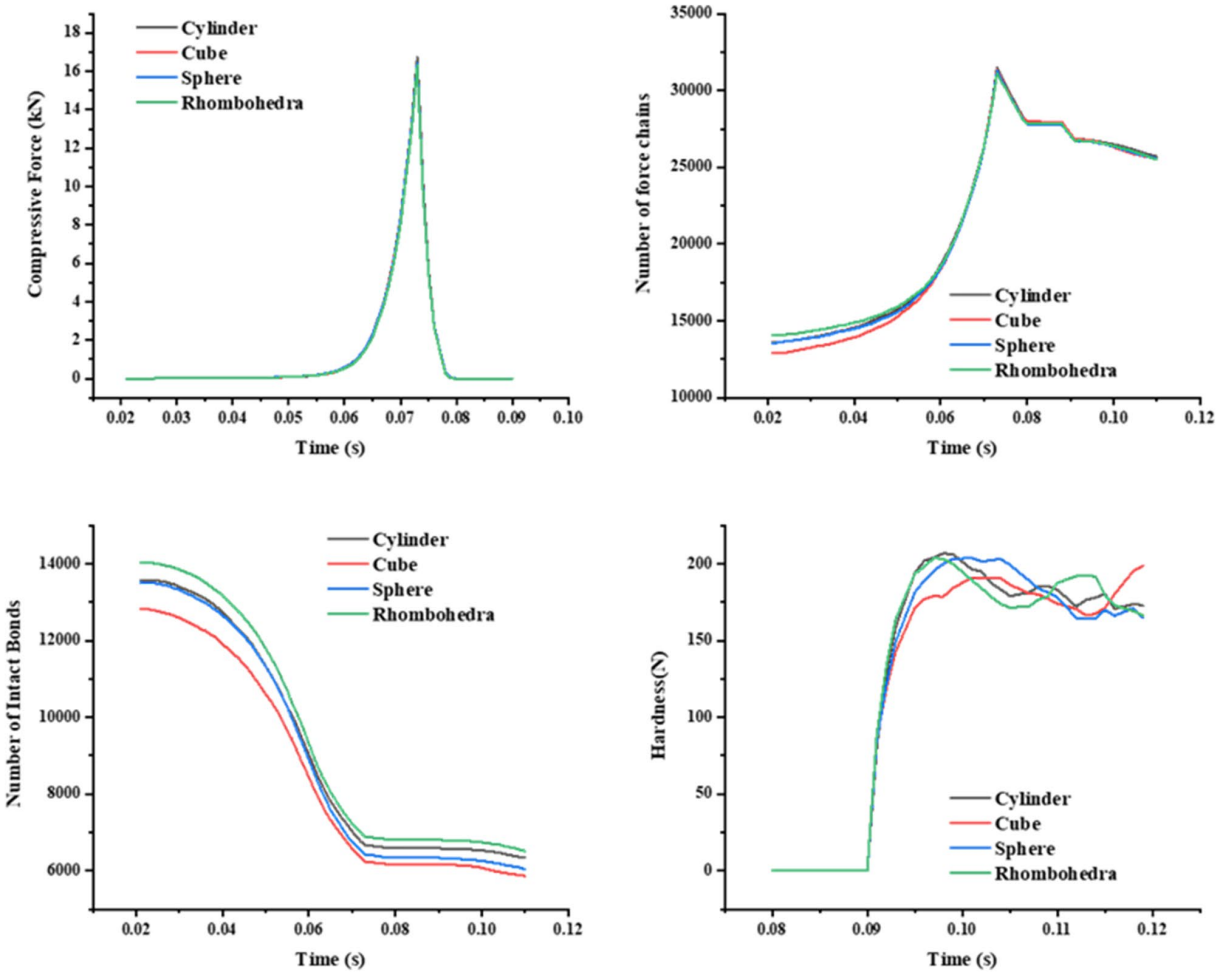
The relation curve between micromechanics behavior and time of the special-shaped clusters formed by single spheres.

This research further explored the mechanism of the impact of special-shaped clusters (spheres, cylinders, cubes, and rhombohedra) on the micromechanics of the tablets (Table 4). The curves of compressive force, force chain contact bonds and hardness with time for tablets formed by special-shaped clusters were extracted (Fig. 16). The results indicate that the change of cluster shape does not affect the micromechanical behavior of the tablets. This may be due to the fact that the special-shaped clusters are all composed of mono-spherical particles and the tablets eventually consist of mono-spherical particles with no change in the inter-particle contact points and contact areas. Therefore, the hardness of the tablets is not affected.

**Table 4:**
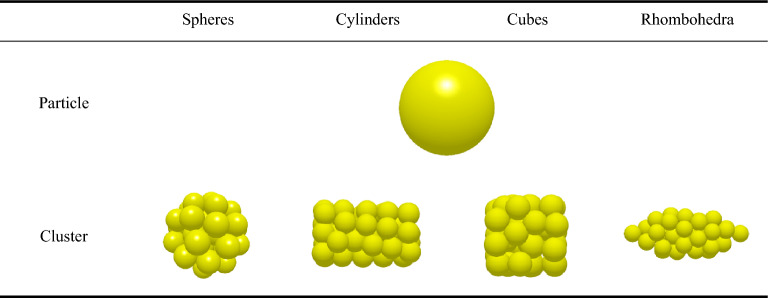
Special-shaped clusters built by single spheres.

## Conclusion

According to the compaction characteristics, the whole compaction process is divided into three stages: rearrangement stage, breakage stage and elastic–plastic deformation stage. The micromechanics behavior of the clusters at each stage are described. The micromechanics behavior in the rearrangement stage is no significant change, the clusters basically does not breakage. In the broken stage, the clusters is continuously breakage, particle fragments fill the gaps, resulting in a rapid decrease in the porosity of the system. In the elastic–plastic deformation stage, the compressive force increases exponentially, and the tablet structure is stable and basically no longer breakage.

A more comprehensive understanding of the respective breakage of the bimodal mixtures at the characteristic points were enlarged and scrutinized. At the rearrangement stage, clusters remain in their original state. In the breakage stage, the PGS clusters breakage first. The MCC clusters gradually breakage in the elastic–plastic deformation stage. This indicated that there was a difference in the breakage time between the elastic excipient PGS and the plastic excipient MCC during compression. This provides some help to understand the evolution pattern of elastic–plastic excipients when they are compressed into tablets and to reveal the formation mechanism of tablets from a microscopic perspective.

With the deformation of the tablet, force chains progressively concentrated into two symmetrical arch-shaped chains. As the tablet deformed, the curvature of the force chains increased. At failure, cracks emerged near the center of the tablet and expanded along the direction perpendicular to the loading plate, damaging the connections between force chains.

The mechanisms of the effects of special-shaped particles as well as special-shaped clusters on the micromechanics of tablets were investigated. The results indicate that the special-shaped particles are formed by overlapping multiple single spheres, the number of contact points and contact area between the particles are changed, and making it easier to form mechanical interlocks between particles. This resulted into significant differences in the micromechanical properties of the final tablets. In contrast, the special-shape clusters consist of single spheres with a consistent number of contact points and contact area between particles. This resulted into no significant differences in the micromechanical properties of the final tablets.

### Supplementary Information

Supplementary Information.

## Data Availability

All data generated and analyzed during this study are either included in the published article itself (or available from the corresponding author upon reasonable request).
