# Supplementary material for: Evolution characteristics of micromechanics provides insights into the microstructure of pharmaceutical tablets fabricated by bimodal mixtures
Source: Sci Rep. 2023 Nov 20;13:20247. doi: 10.1038/s41598-023-47239-w (PMC10662154; doi:10.1038/s41598-023-47239-w)
Supplement: Supplementary file 1 — Supplementary Information. [file 41598_2023_47239_MOESM1_ESM.docx]

Supplementary Information

Evolution characteristics of micromechanics provides insights into the microstructure of pharmaceutical tablets fabricated by bimodal mixtures

Mengtao Zhao^a^, Anqi Luo^a^, Yu Zhou^a^, Zeng Liu^a^, Yuting Wang^a^, Linxiu Luo^a^, Yanling Jiang^a^, Jingcao Tang^a^, Zheng Lu^a^, Tianbing Guan^a^, Libo Chen^a^, Huimin Sun^b^, Chuanyun Dai^a^

^a^ Chongqing Key Laboratory of Industrial Fermentation Microorganisms, College of Chemistry and Chemical Engineering, Chongqing University of Science and Technology, Chongqing, 401331, China
^b^ NMPA Key Laboratory for Quality Research and Evaluation of Pharmaceutical Excipients,

National Institutes for Food and Drug Control, Beijing 100050, China

^*^Corresponding author. Email address: sunhm@126.com (H. Sun) and [cydai@126.com](mailto:cydai@126.com) (C. Dai); Tel: 86-10-53852486(H. Sun) and 86-23-65022212(C. Dai).

Based on the pre-experiments, the parameters such as Poisson's ratio (A), Shear modulus (B), Static friction coefficient (C), Normal stiffness per unit area (D), Shear stiffness per unit area (E), and Constant pull-off force (F), which have a significant effect on the mechanical behavior of tablets, were selected for parameter calibration in the present study.

**Table S1.** Optimal Latin hypercube test sample and optimization target results (PGS).

| Number | A | B  (MPa) | C | D  (N/m^3^) | E  (N/m^3^) | F  (N) | Hardness-  Compressive force | Volume reduction-Compressive force |
| --- | --- | --- | --- | --- | --- | --- | --- | --- |
| 1 | 0.3034 | 4.45 | 0.238 | 59.03 | 1 | -0.00759 | 774.1 | 1147.32 |
| 2 | 0.3517 | 1.28 | 0.155 | 76.1 | 35.14 | -0.00672 | 724.76 | 517.83 |
| 3 | 0.3586 | 2.66 | 0.266 | 41.97 | 96.59 | -0.00586 | 924.53 | 1028.38 |
| 4 | 0.2345 | 2.1 | 0.348 | 96.59 | 14.66 | -0.00862 | 508.5 | 851.71 |
| 5 | 0.2069 | 3.34 | 0.597 | 24.9 | 93.17 | -0.00655 | 862.7 | 1167.14 |
| 6 | 0.2 | 1.55 | 0.845 | 55.62 | 41.97 | -0.00793 | 421.22 | 832.54 |
| 7 | 0.2897 | 4.86 | 0.321 | 28.31 | 52.21 | -0.00517 | 996.72 | 1231.61 |
| 8 | 0.37 | 3.21 | 0.21 | 65.86 | 55.62 | -0.00983 | 504.62 | 1017.21 |
| 9 | 0.34 | 4.31 | 0.79 | 69.28 | 21.48 | -0.00948 | 558.45 | 1353.91 |
| 10 | 0.25 | 1.14 | 0.293 | 45.38 | 79.52 | -0.00845 | 314.99 | 302.76 |
| 11 | 0.39 | 1.83 | 0.734 | 72.69 | 65.86 | -0.00810 | 528.48 | 1086.72 |
| 12 | 0.28 | 2.52 | 0.128 | 1.00 | 28.31 | -0.00741 | 794.51 | 630.82 |
| 13 | 0.23 | 2.24 | 0.376 | 52.21 | 24.90 | -0.00500 | 952.54 | 951.14 |
| 14 | 0.22 | 4.59 | 0.679 | 79.52 | 38.55 | -0.00690 | 814.88 | 1349.71 |
| 15 | 0.26 | 3.48 | 0.762 | 11.24 | 11.24 | -0.00707 | 805.25 | 1297.97 |
| 16 | 0.34 | 3.90 | 0.9 | 48.79 | 62.45 | -0.00534 | 1040.64 | 1495.91 |
| 17 | 0.28 | 4.17 | 0.183 | 14.66 | 89.76 | -0.00897 | 699.92 | 1040.45 |
| 18 | 0.21 | 3.62 | 0.459 | 35.14 | 31.72 | -0.01000 | 449.22 | 1138.74 |
| 19 | 0.24 | 3.76 | 0.1 | 82.93 | 69.28 | -0.00724 | 890.87 | 958.86 |
| 20 | 0.26 | 2.93 | 0.652 | 93.17 | 82.93 | -0.00966 | 412.5 | 1203.36 |
| 21 | 0.32 | 2.38 | 0.817 | 86.34 | 4.41 | -0.00638 | 814.23 | 1199.5 |
| 22 | 0.27 | 1.97 | 0.624 | 89.76 | 86.34 | -0.00569 | 850.07 | 1038.18 |
| 23 | 0.39 | 1.69 | 0.403 | 4.41 | 72.69 | -0.00879 | 464.13 | 895.16 |
| 24 | 0.30 | 3.07 | 0.872 | 21.48 | 76.10 | -0.00931 | 420.52 | 1271.26 |
| 25 | 0.32 | 5.00 | 0.569 | 62.45 | 100.00 | -0.00776 | 681.88 | 1393.88 |
| 26 | 0.3793 | 4.72 | 0.514 | 7.83 | 45.38 | -0.00828 | 670.05 | 1343.4 |
| 27 | 0.331 | 1.41 | 0.541 | 38.55 | 7.83 | -0.00914 | 308.18 | 779.56 |
| 28 | 0.3724 | 4.03 | 0.431 | 100 | 48.79 | -0.00621 | 880.86 | 1248.28 |
| 29 | 0.4 | 2.79 | 0.486 | 31.72 | 18.07 | -0.00552 | 927.53 | 1200.35 |
| 30 | 0.3103 | 1 | 0.707 | 18.07 | 59.03 | -0.00603 | 600.13 | 602.56 |

**Table S2.** Optimal Latin hypercube test sample and optimization target results (MCC).

| Number | A | B  (MPa) | C | D  (N/m^3^) | E  (N/m^3^) | | F  (N) | | Hardness - Compressive force | | Volume reduction-Compressive force | |
| --- | --- | --- | --- | --- | --- | --- | --- | --- | --- | --- | --- | --- |
| 1 | 0.3379 | 79.52 | 0.707 | 1.93 | 9.07 | | -0.0329 | | 321.86 | | 272.43 | |
| 2 | 0.3103 | 45.38 | 0.1 | 6.59 | 7.83 | | -0.0081 | | 684.6 | | 99.94 | |
| 3 | 0.3586 | 14.66 | 0.817 | 2.24 | 3.48 | | -0.0314 | | 104.74 | | 41.66 | |
| 4 | 0.3172 | 41.97 | 0.128 | 9.69 | 2.55 | | -0.0345 | | 286.41 | | 96.16 | |
| 5 | 0.2414 | 69.28 | 0.238 | 1 | 3.79 | | -0.019 | | 527.61 | | 173.95 | |
| 6 | 0.2 | 65.86 | 0.403 | 4.1 | 10 | | -0.0283 | | 373.69 | | 192.24 | |
| 7 | 0.3655 | 18.07 | 0.266 | 3.48 | 8.76 | | -0.0376 | | 0.58 | | -0.46 | |
| 8 | 0.3793 | 20 | 0.486 | 8.14 | 5.34 | | -0.0205 | | 112.3 | | 74.38 | |
| 9 | 0.2759 | 40 | 0.597 | 4.41 | 7.21 | | -0.005 | | 579.06 | | 179.05 | |
| 10 | 0.2897 | 28.31 | 0.459 | 5.34 | 1.62 | | -0.0066 | | 668.3 | | 113.04 | |
| 11 | 0.2552 | 35.14 | 0.541 | 1.31 | 5.97 | | -0.05 | | 85.15 | | 140.6 | |
| 12 | 0.2276 | 24.9 | 0.624 | 9.38 | 7.52 | | -0.0159 | | 449.62 | | 99.74 | |
| 13 | 0.2828 | 11.24 | 0.569 | 2.55 | 8.14 | | -0.0112 | | 472.1 | | -52.05 | |
| 14 | 0.331 | 93.17 | 0.734 | 3.17 | 1.93 | | -0.0267 | | 430.88 | | 280.31 | |
| 15 | 0.3241 | 89.76 | 0.155 | 4.72 | 6.28 | | -0.0407 | | 327.03 | | 187.83 | |
| 16 | 0.4 | 55.62 | 0.376 | 1.62 | 5.66 | | -0.0128 | | 607.19 | | 210.33 | |
| 17 | 0.3448 | 76.1 | 0.514 | 9.07 | 9.69 | | -0.0252 | | 417.58 | | 238.31 | |
| 18 | 0.2966 | 62.45 | 0.79 | 10 | 2.24 | | -0.0236 | | 462.37 | | 247.85 | |
| 19 | 0.2069 | 48.79 | 0.845 | 3.79 | 4.1 | | -0.0221 | | 419.5 | | 215.79 | |
| 20 | 0.3931 | 59.03 | 0.652 | 6.9 | 4.72 | | -0.0469 | | 132.62 | | 239.77 | |
| 21 | 0.2345 | 72.69 | 0.431 | 5.66 | 1 | | -0.0453 | | 140.07 | | 210.02 | |
| 22 | 0.2621 | 38.55 | 0.321 | 8.76 | 8.45 | | -0.0484 | | 37.01 | | 128.27 | |
| 23 | 0.3517 | 31.72 | 0.21 | 2.86 | 1.31 | | -0.036 | | 187.01 | | 90.31 | |
| 24 | 0.3724 | 52.21 | 0.9 | 5.97 | 6.59 | | -0.0097 | | 652.08 | | 257.59 | |
| 25 | 0.2138 | 82.93 | 0.293 | 8.45 | 4.41 | | -0.0174 | | 557.58 | | 200.6 | |
| 26 | 0.3862 | 86.34 | 0.348 | 7.83 | 3.17 | | -0.0143 | | 604.68 | | 221.46 | |
| 27 | 0.2483 | 96.59 | 0.762 | 7.21 | 6.9 | | -0.0422 | | 196.3 | | 269.72 | |
| 28 | 0.3034 | 21.48 | 0.872 | 6.28 | 9.38 | -0.0391 | | 36.01 | | 114.58 | |  |

**
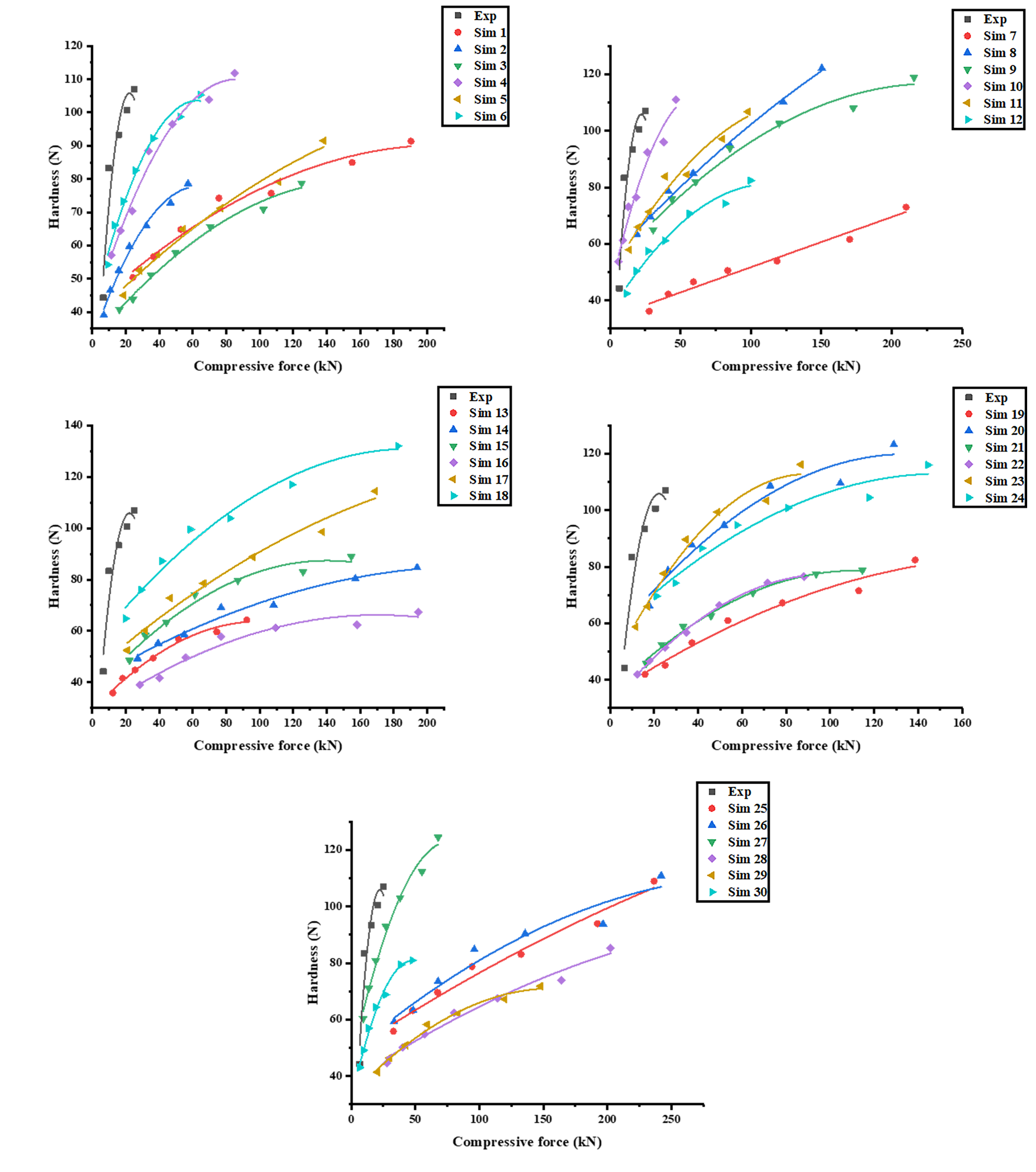
**

**Fig. S1** The curve of hardness versus compressive force for experimental and DEM simulations (PGS).


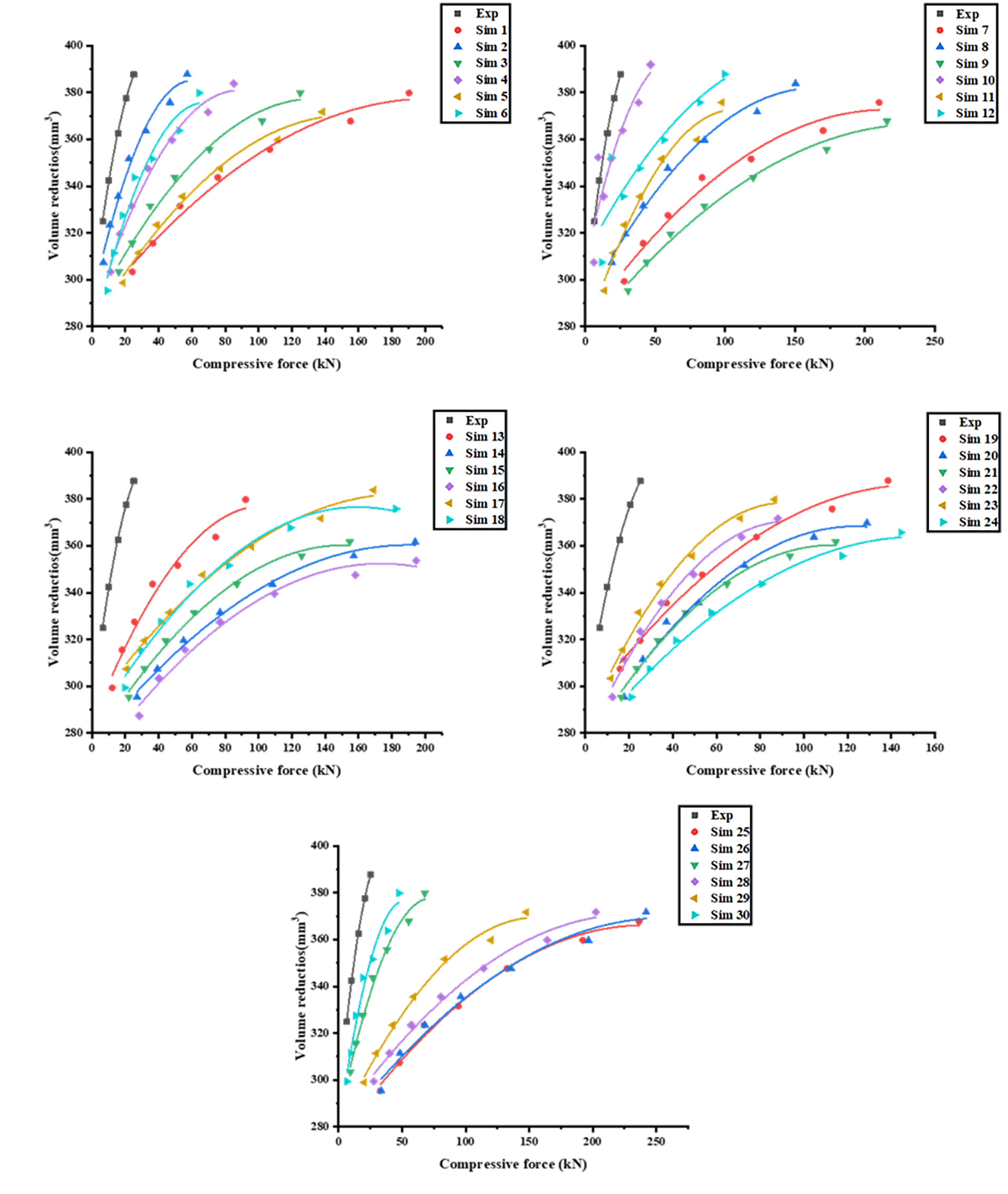


**Fig. S2** The curve of volume reduction versus compressive force for experimental and DEM simulations (PGS).


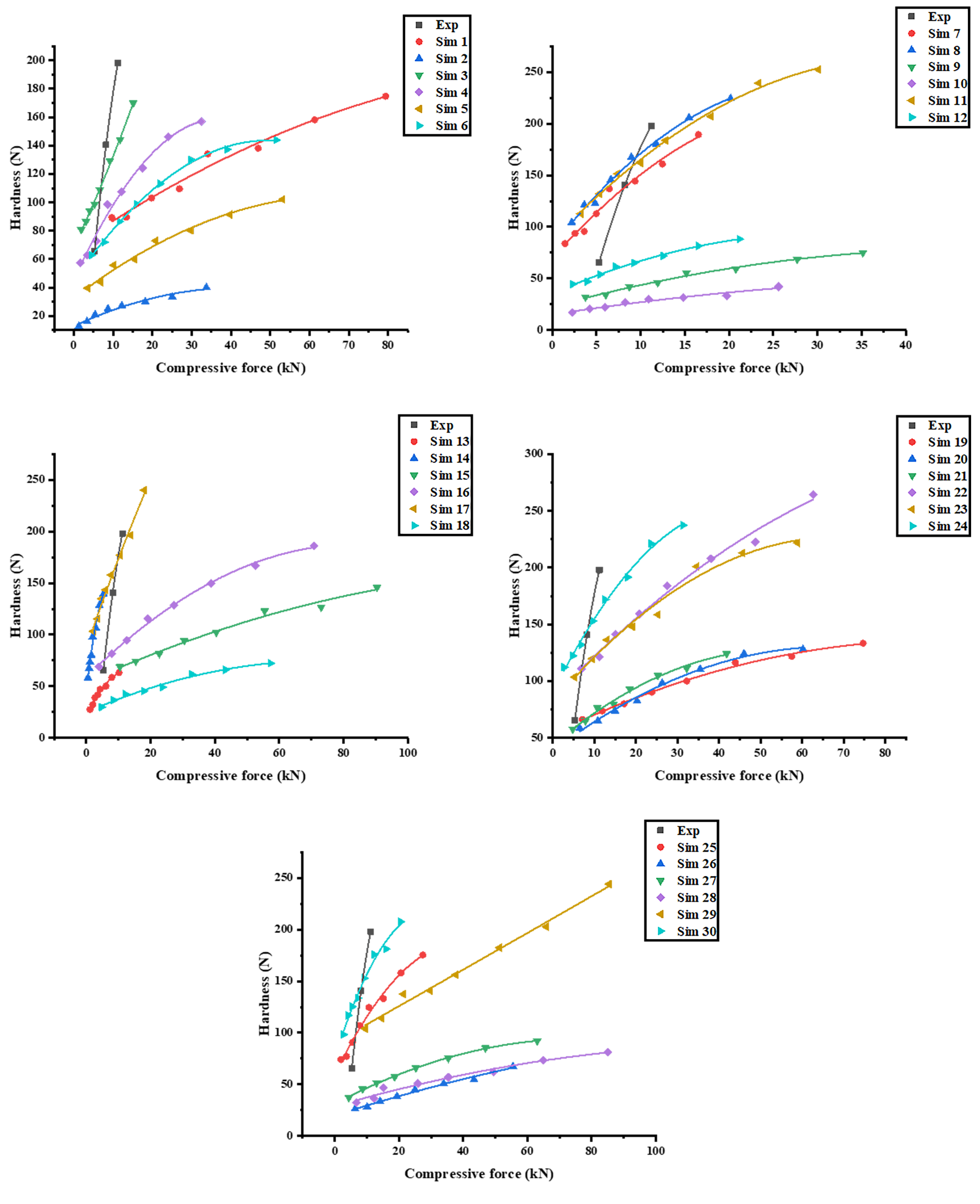


**Fig. S3** The curve of hardness versus compressive force for experimental and DEM simulations (MCC).


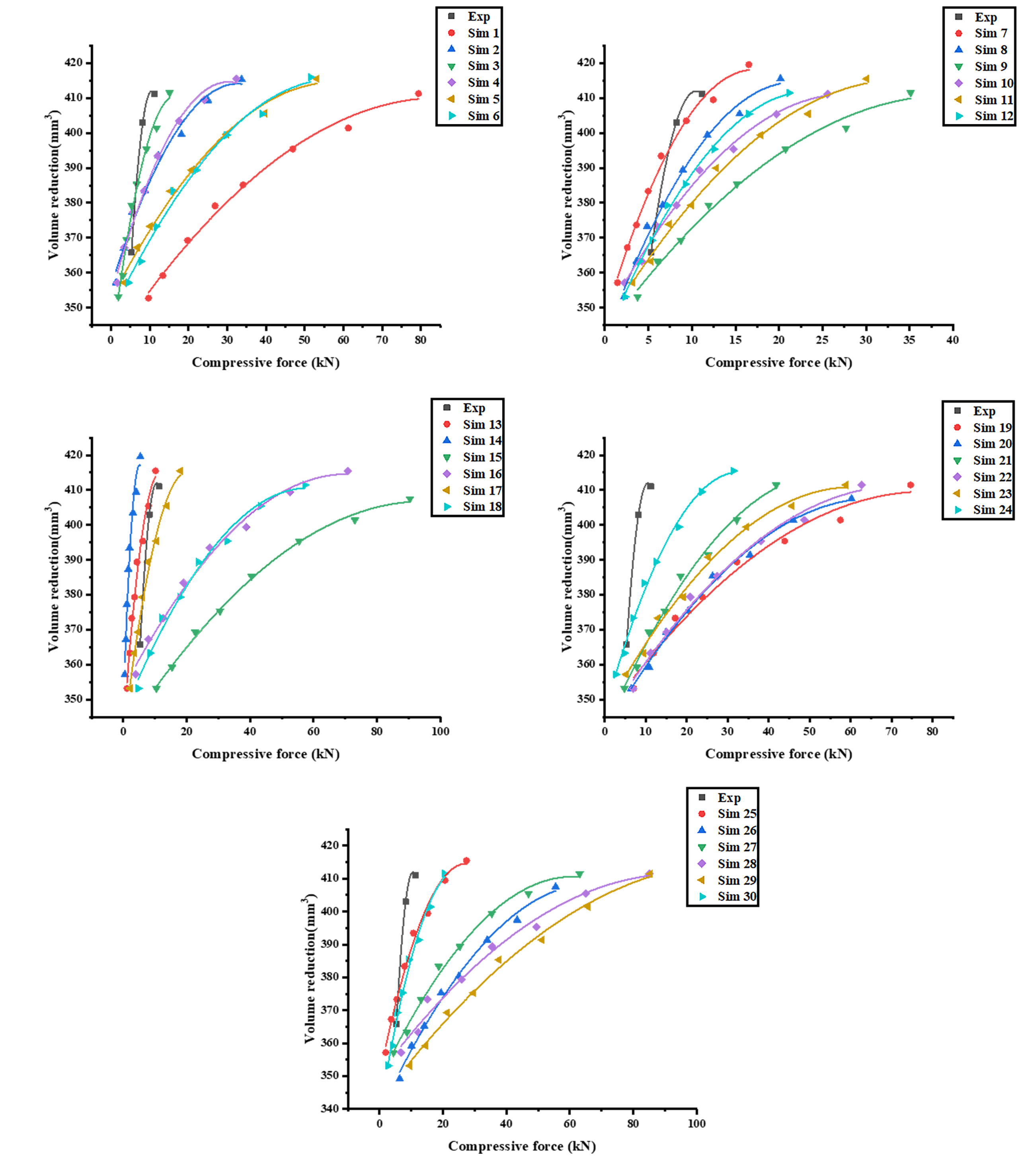


**Fig. S4** The curve of volume reduction versus compressive force for experimental and DEM simulations (MCC).


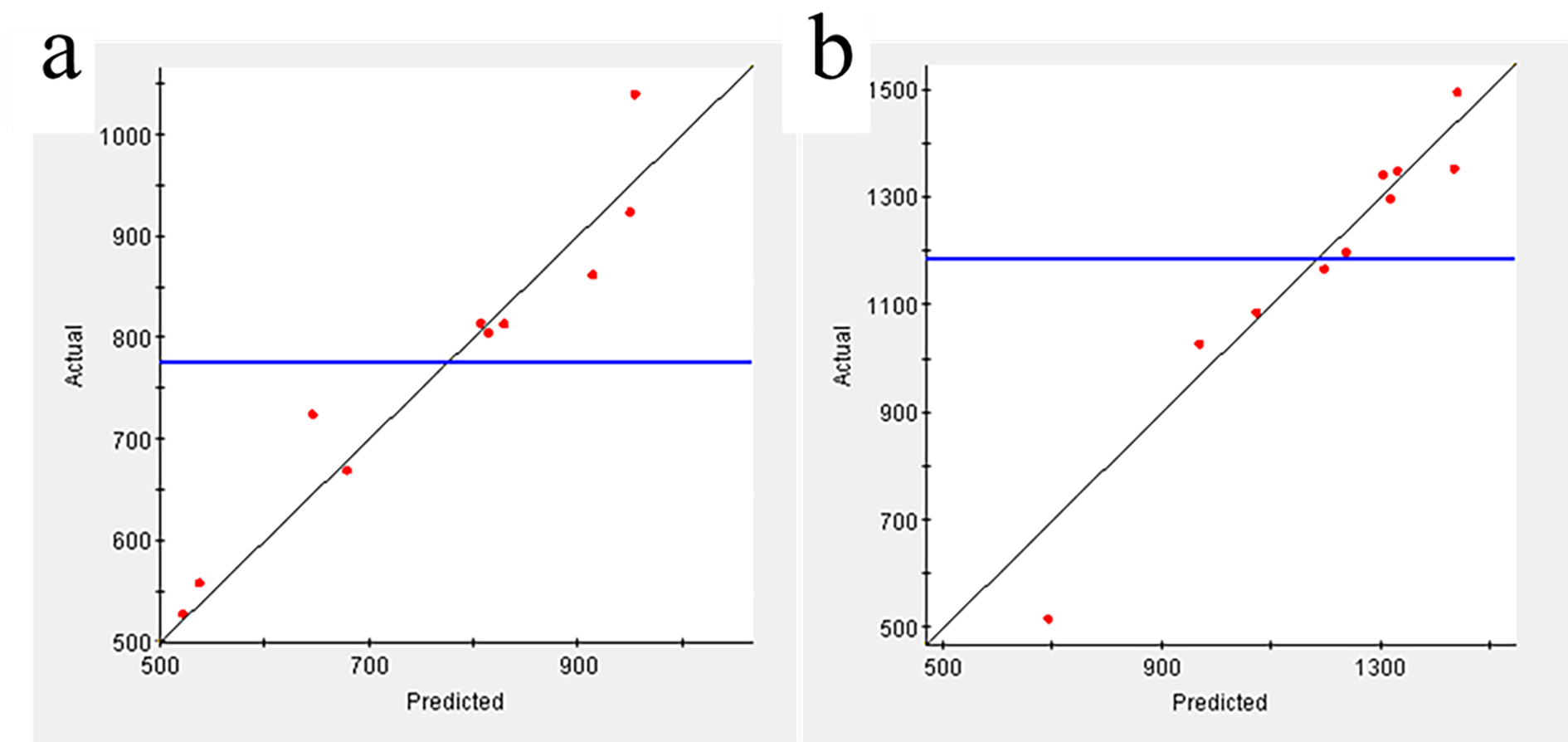


**Fig. S5** Kriging model accuracy validation results (PGS).

(a: Hardness-Compressive force; b: Volume reduction-Compressive force)


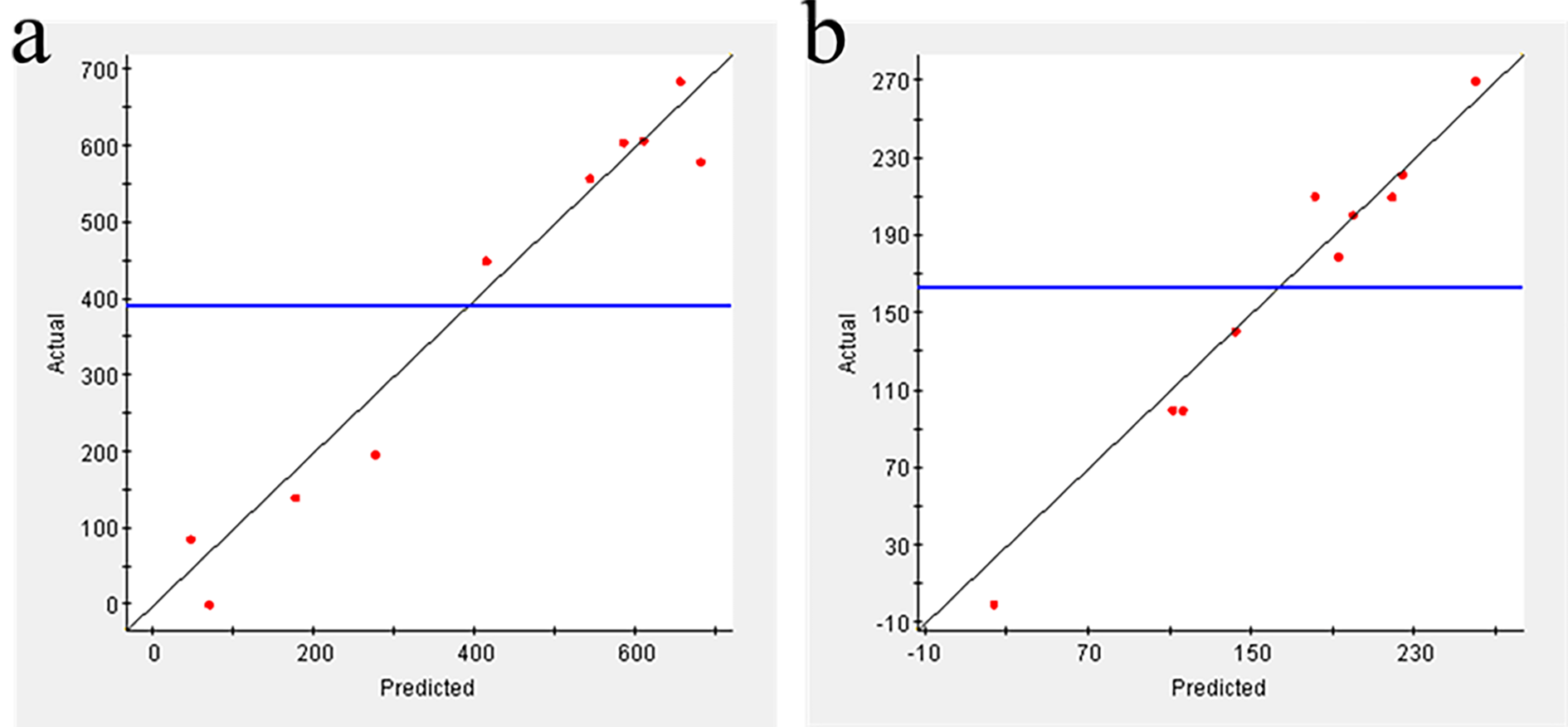


**Fig. S6** Kriging model accuracy validation results (MCC).

(a: Hardness-Compressive force; b: Volume reduction-Compressive force)
